# Angiotensin II induces connective tissue growth factor expression in human hepatic stellate cells by a transforming growth factor *β*-independent mechanism

**DOI:** 10.1038/s41598-017-08334-x

**Published:** 2017-08-10

**Authors:** Ao Li, Jingyao Zhang, Xiaoxun Zhang, Jun Wang, Songsong Wang, Xiao Xiao, Rui Wang, Peng Li, Yitao Wang

**Affiliations:** 1 College of Pharmacy and Bioengineering, Chongqing University of Technology, Chongqing, 400054 China; 2 State Key Laboratory of Quality Research in Chinese Medicine, Institute of Chinese Medical Sciences, University of Macau, Macau, 999078 China

## Abstract

Angiotensin II (Ang II) promotes hepatic fibrosis by increasing extracellular matrix (ECM) synthesis. Connective tissue growth factor (CTGF) plays a crucial role in the pathogenesis of hepatic fibrosis and emerges as downstream of the profibrogenic cytokine transforming growth factor-*β* (TGF-*β*). We aimed to investigate the molecular events that lead from the Ang II receptor to CTGF upregulation in human hepatic stellate cells, a principal fibrogenic cell type. Ang II produced an early, AT_1_ receptor-dependent stimulation of CTGF expression and induced a rapid activation of PKC and its downstream p38 MAPK, thereby activating a nuclear factor-*κ*B (NF-*κ*B) and Smad2/3 cross-talk pathway. Chemical blockade of NF-*κ*B and Smad2/3 signaling synergistically diminished Ang II-mediated CTGF induction and exhibited an additive effect in abrogating the ECM accumulation caused by Ang II. Furthermore, we demonstrated that CTGF expression was essential for Ang II-mediated ECM synthesis. Interestingly, the ability of dephosphorylated, but not phosphorylated JNK to activate Smad2/3 signaling revealed a novel role of JNK in Ang II-mediated CTGF overexpression. These results suggest that Ang II induces CTGF expression and ECM accumulation through a special TGF-*β*-independent interaction between the NF-*κ*B and Smad2/3 signals elicited by the AT_1_/PKC*α*/p38 MAPK pathway.

## Introduction

Hepatic fibrosis is an important pathological feature of many liver diseases, which is characterized by fibroblast proliferation and excessive accumulation of extracellular matrix (ECM) components^1^. Hepatic stellate cells (HSCs) are the major cell type involved in regulating ECM deposition in liver upon injury^2^. Following injury, HSCs differentiate into myofibroblast-like cells and produce a variety of mediators which participate in regulating the development of hepatic fibrosis^3^. Recently, HSCs have been found as the major cellular source of CTGF in the liver^4^. CTGF not only triggers and perpetuates HSC activation *via* an autocrine or paracrine mechanism, but also synergizes with transforming growth factor-*β* (TGF-*β*) to stimulate ECM synthesis in activated HSCs^5–7^. In a mice model of hepatic fibrosis, blockade of CTGF synthesis by CTGF antisense oligonucleotide injection diminishes TGF-*β*1 and type I collagen mRNA transcription levels^8^. Owing to these properties, CTGF may represent an attractive therapeutic target for hepatic fibrosis.

During hepatic wound healing, several substances, especially endothelin-1 and platelet-derived growth factor, can stimulate the secretion of mature angiotensin II (Ang II) stored in activated HSCs^9^. This locally produced Ang II has proinflammatory and profibrogenic effects on HSCs^10^. Ang II as well as other components of the renin-angiotensin system (RAS) is significantly up-regulated both in serum and liver tissue from patients with chronic liver diseases^11^. In experimental models of hepatic fibrosis, angiotensin-converting enzyme inhibitors (ACE-I) and/or Ang II type 1 receptor (AT_1_) blocker significantly inhibited the upregulation of TGF-*β* expression and HSC activation^12, 13^. These data suggest that Ang II has a central role in the pathogenesis and progression of tissue remodeling and fibrogenesis in the liver^14^. Moreover, a cross-talk between Ang II and CTGF in cardiovascular and kidney diseases has been previously demonstrated^15, 16^. However, the relationship between Ang II and CTGF in HSCs remains uncertain.

It is well known that most of the pathophysiological biological effects elicited by Ang II are mediated by AT_1_. Ang II, through binding to cognate receptor AT_1_, triggers activation of protein kinase C (PKC), a superfamily at least including 12 different closely related serine/threonine kinases^17^. PKC activation induces phosphorylation and activation of mitogen-activated protein kinase (MAPK) family, including c-Jun N-terminal kinase (JNK), extracellular signal-regulated kinase 1/2 (ERK1/2) and p38 MAPK in HSCs, which are among the major mediators of the profibrotic effects induced by Ang II, leading to fibrotic-related gene transcription and connective tissue formation in fibrotic disorders^18^.

Because a specific TGF-*β*-responsive element (T*β*RE) is present in human and murine CTGF promoter region^19^, the synthesis of CTGF induced by Ang II is mainly regulated at transcriptional level in a TGF-*β*-dependent manner in fibrotic tissues^20, 21^. However, some investigators have reported that in vascular smooth muscle cells, Ang II activates the Smad signaling pathway *via* TGF-*β*-independent p38 MAPK activation, and this links to vascular fibrosis^22^. In tubular epithelial cells lacking the *TGF-β* gene, Ang II upregulates CTGF expression through an AT_1_-mediated ERK1/2 and p38 MAPK cross-talk pathway, which is also TGF-*β* independent^16^. These observations raise the possibility that additional signaling mechanisms independent of TGF-*β* may be required for Ang II-induced CTGF expression. In addition to Smad, researchers also reveal that there are several consensus sequences of nuclear factor kappa B (NF-*κ*B) on CTGF promoter^23^. NF-*κ*B inhibition leads to significant downregulation of CTGF expression in cardiac fibroblasts^24^. Thus, we postulated that in addition to the TGF-*β*-dependent mechanism, Ang II might induce upregulation of CTGF expression *via* several TGF-*β*-independent signaling pathways in HSCs. The current study was initiated to investigate a mechanism by which the NF-*κ*B and Smad2/3 pathways interact and to further delineate a role for the interaction in Ang II stimulation of CTGF expression; and to evaluate the hypothesis that CTGF could be a key contributor to Ang II-induced ECM accumulation in human HSCs.

## Results

### Ang II-induced early CTGF expression is independent of TGF-*β*1 generation or activation in LX-2 cells

**Figure 1:**
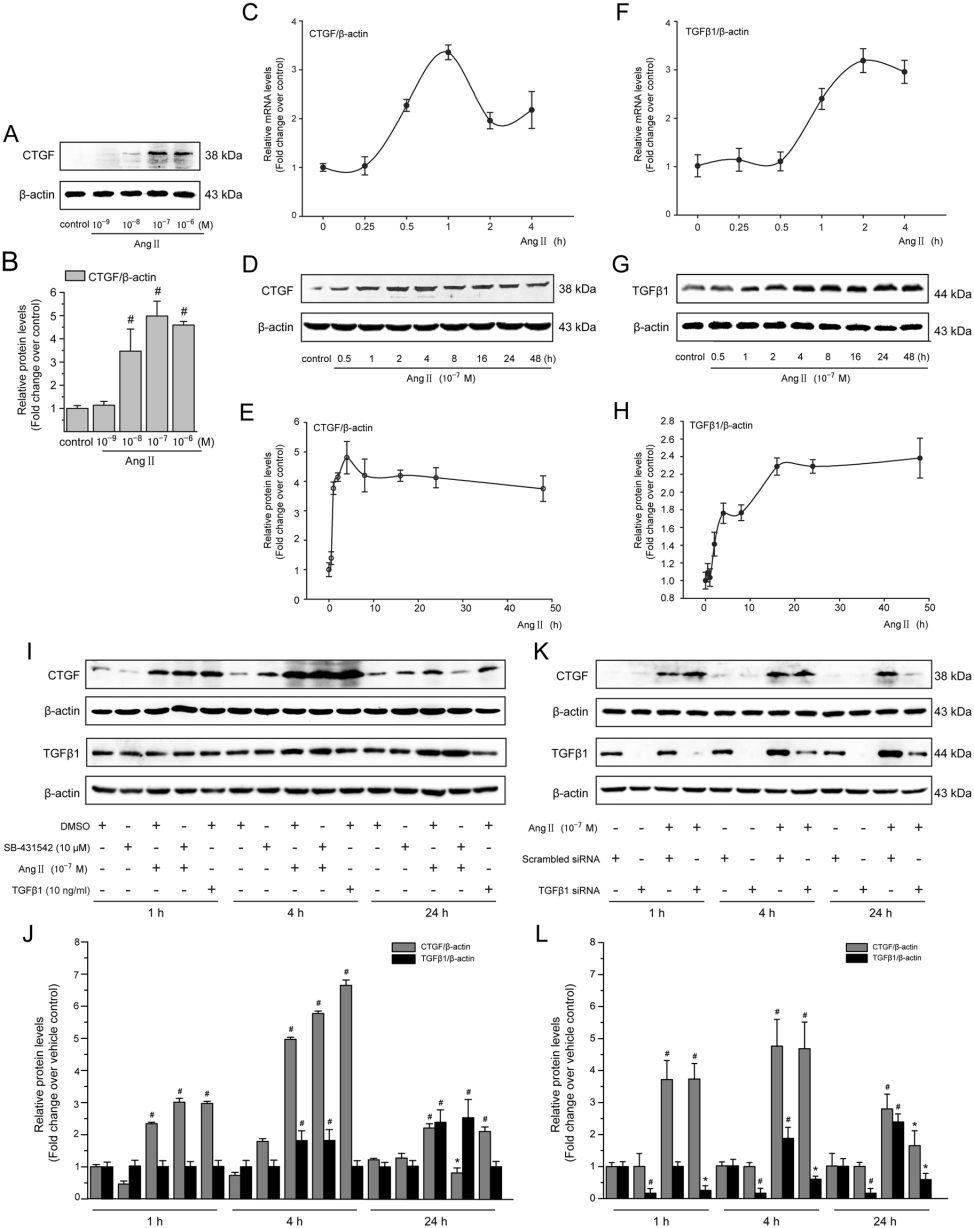
Ang II induces a rapid upregulation of CTGF expression independently of TGF-*β* in LX-2 cells. (**A**) Serum-starved LX-2 cells were stimulated with Ang II (10^−8^–10^−6^ M) for 4 h. Whole cell lysates were immunoblotted with CTGF antibody and a representative immunoblot band of CTGF from 3 independent experiments is shown. *β*-Actin was used as an internal control for equal protein loading. (**B**) Results of total CTGF expression were obtained from densitometric analysis and expressed as ratio CTGF/*β*-actin, as *n*-fold increase over that in unstimulated control cells. (**C,F**) Serum-starved LX-2 cells were exposed to Ang II (10^−7^ M) for 0, 0.25, 0.5, 1, 2 and 4 h. The mRNA expression of the *TGF-β1* and *CTGF* gene was measured by the qRT-PCR method as described in the Materials and Methods section. (**D,G**) Serum-starved LX-2 cells were exposed to Ang II (10^−7^ M) for 0, 0.5, 1, 2, 4, 8, 16, 24 and 48 h. Whole cell lysates were prepared and immunoblotted with anti-CTGF or anti-TGF-*β*1 antibodies, respectively. Anti-*β*-actin antibody was used to demonstrate equal protein loading. Similar results were observed in 3 independent experiments, and representative immunoblot bands of CTGF and TGF-*β*1 are shown. (**E,H**) Quantitative determination of CTGF and TGF-*β*1 protein levels at various time points as indicated, which were converted to arbitrary densitometric units, normalized by the value of *β*-actin and finally expressed as *n*-fold change over that of unstimulated control cells (defined as 1.0). (**I,J**) TGF-*β* signaling was blocked by pretreatment for 1 h with SB-431542 (a TGF-*β* receptor kinase inhibitor; 10^−5^ M), (**K,L**) or by transfection with TGF-*β*1 siRNA, before incubation with or without Ang II (10^−7^ M) for 1, 4 and 24 h. The CTGF and TGF-*β*1 protein levels were analyzed by Western blotting. As a positive control of CTGF production, TGF-*β*1 (10 ng/mL) was used. Data are presented as mean ± SD of 3 independent experiments. ^#^ *P* < 0.05 *versus* unstimulated, or vehicle- or scrambled siRNA-treated control cells; ^*^ *P* < 0.05 *versus* Ang II- or scrambled siRNA + Ang II-treated cells.

Incubation of LX-2 cells with Ang II (10^−8^–10^−6^ M) for 4 h induced a marked increase of CTGF protein, with a maximal peak at 10^−7^ M (Fig. 1A,B). *CTGF* transcript levels started increasing sharply from 0.25 to 0.5 h upon treatment with Ang II (10^−7^ M) and peaked at 1 h (3.4 fold) and remained markedly higher than the initial levels until the end of 4 h simulation (Fig. 1C). Similarly, Ang II-mediated induction of CTGF protein occurred within 0.5 h and reached peak (4.8-fold) after 4 h of Ang II incubation (Fig. 1D,E). However, longer periods of incubation (8–48 h post-Ang II treatment) did not further increase CTGF protein level. Thus, subsequent experiments were carried out with Ang II (10^−7^ M) stimulation for 0–4 h.

Previous studies demonstrated that Ang II induces CTGF expression predominantly through the TGF-*β*-dependent pathways^20, 21^. To determine whether upregulation of CTGF expression in LX-2 cells was mediated by endogenous TGF-*β* synthesis or directly induced by Ang II, we examined the mRNA and protein levels of TGF-*β*1 at various time points. Ang II did not increase TGF-*β*1 mRNA levels in the first 0.5 h, but this gene was upregulated at 1 h and remained elevated until 4 h (Fig. 1F). Consistent with the mRNA results, Ang II did not significantly influence TGF-*β*1 protein abundance within 0.25–2 h of incubation (Fig. 1G,H). Protein level of TGF-*β*1 was significantly increased at 4 h, whereas it reached to a maximum 48 h after Ang II incubation. These data indicate that a robust induction of CTGF mRNA and protein by Ang II appears before the increase in TGF-*β*1 expression.

To further elucidate Ang II-induced CTGF upregulation is mediated by a TGF-*β*-independent mechanism, two different strategies to block TGF-*β* signaling: SB-431542, an inhibitor of TGF-*β* type I receptor (T*β*R I) kinase activity, and a specific siRNA for TGF-*β*1, were used. In LX-2 cells, pharmacological inhibition of T*β*R I activity by SB-431542 or knockdown of TGF-*β*1 by siRNA transfection did not modify Ang II-dependent CTGF induction after 1 and 4 h of incubation (Fig. 1I–L). These data show that Ang II induces early CTGF protein expression independent of endogenous TGF-*β* generation or activation. In contrast, blocking TGF-*β* signaling by SB-431542 treatment or knockdown of TGF-*β*1 by siRNA significantly reduced the Ang II-induced CTGF protein levels after co-treatment for 24 h, indicating that endogenous and exogenous TGF-*β* is involved in long-term CTGF induction by Ang II. In addition, the results obtained by Western blotting show that the inhibition of TGF-*β*1 signaling by SB-431542 did not have any effect on the early (1 or 4 h) and late (24 h) TGF-*β*1 expression, regardless of whether cells were incubated with Ang II.

### Involvement of AT_1_ in Ang II-induced CTGF expression in LX-2 cells

**Figure 2:**
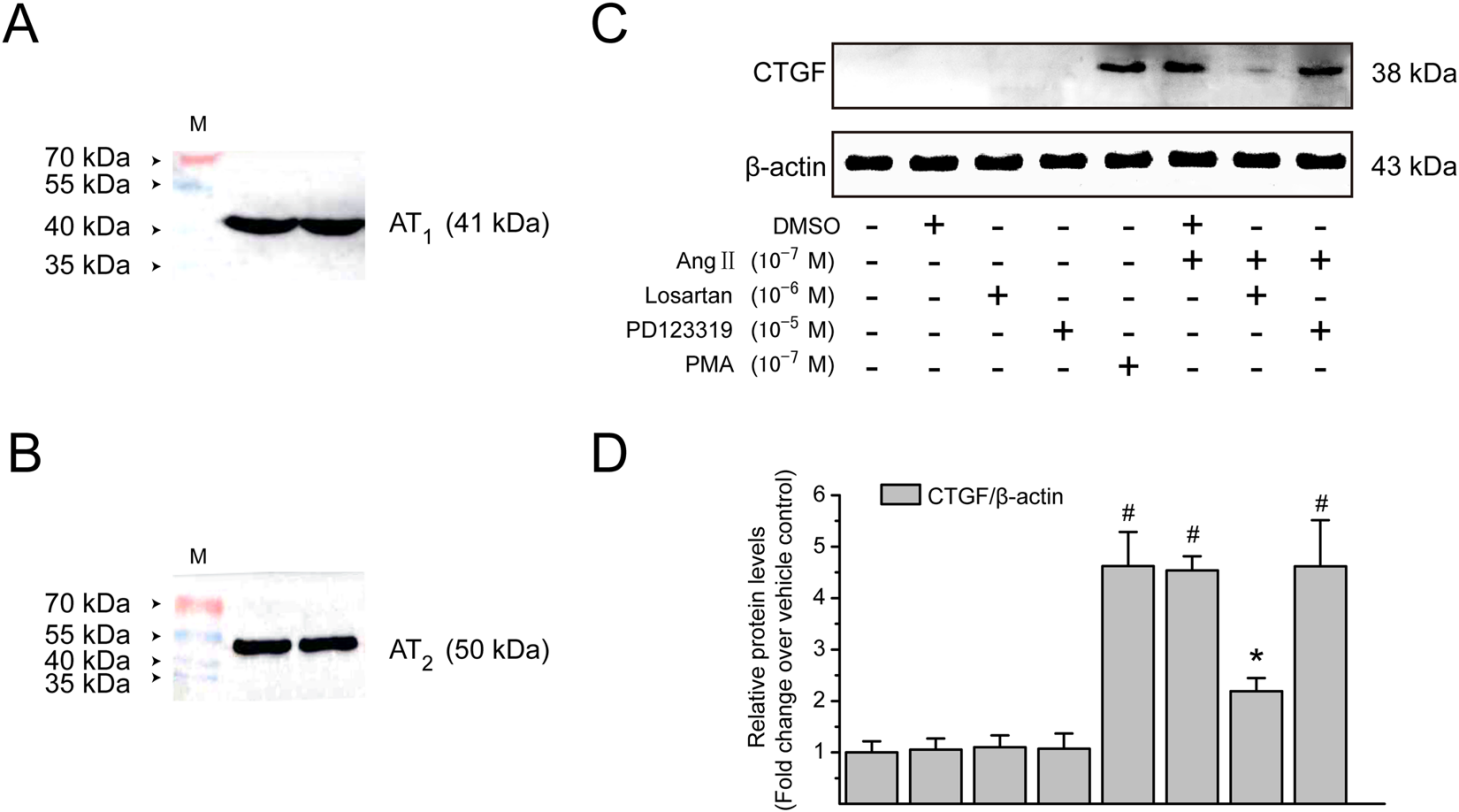
Ang II induces CTGF protein expression *via* an AT_1_-dependent mechanism in LX-2 cells. (**A,B**) Immunoblotting analysis was performed using whole cell lysates from unstimulated control cells to identify AT_1_ and AT_2_. (**C**) Serum-starved LX-2 cells were preincubated with losartan (an AT_1_ receptor antagonist; 10^−6^ M) or PD123319 (an AT_2_ receptor antagonist; 10^−5^ M) for 1 h and then incubated with or without Ang II (10^−7^ M) for 4 h. PMA (10^−7^ M; a PKC activator) was used as a positive control. Cell extracts were collected and subjected to immunoblotting analysis using anti-CTGF antibody. *β*-Actin was used as an internal control for equal loading of total protein amounts. The experiments were repeated thrice with similar results and representative immunoblot bands are shown. (**D**) The protein levels of CTGF were converted to arbitrary densitometric units, normalized by the value of *β*-actin and expressed relative to the level of CTGF in the vehicle-treated cells (defined as 1-fold). The results are shown as mean ± SD of 3 independent experiments. ^#^ *P* < 0.05 *versus* vehicle-treated control cells; ^*^ *P* < 0.05 *versus* Ang II-treated cells.

Two Ang II receptor subtypes, AT_1_ and AT_2_, are expressed on HSCs (Fig. 2A,B) and play a pivotal role in hepatic fibrosis development. To clarify which receptor subtype mediates Ang II-induced CTGF expression, LX-2 cells were incubated with either a specific AT_1_ antagonist losartan or an AT_2_ antagonist PD123319, prior to stimulation with Ang II (10^−7^ M). Induction of CTGF protein by Ang II was completely abolished by losartan, but not by PD123319 (Fig. 2C,D), which demonstrates that Ang II induces CTGF protein accumulation by initiating signaling through the AT_1_. Interestingly, a PKC activator, PMA, also led to upregulation of CTGF expression in LX-2 cells.

### Ang II-induced PKC activation depends on AT_1_ in LX-2 cells

**Figure 3:**
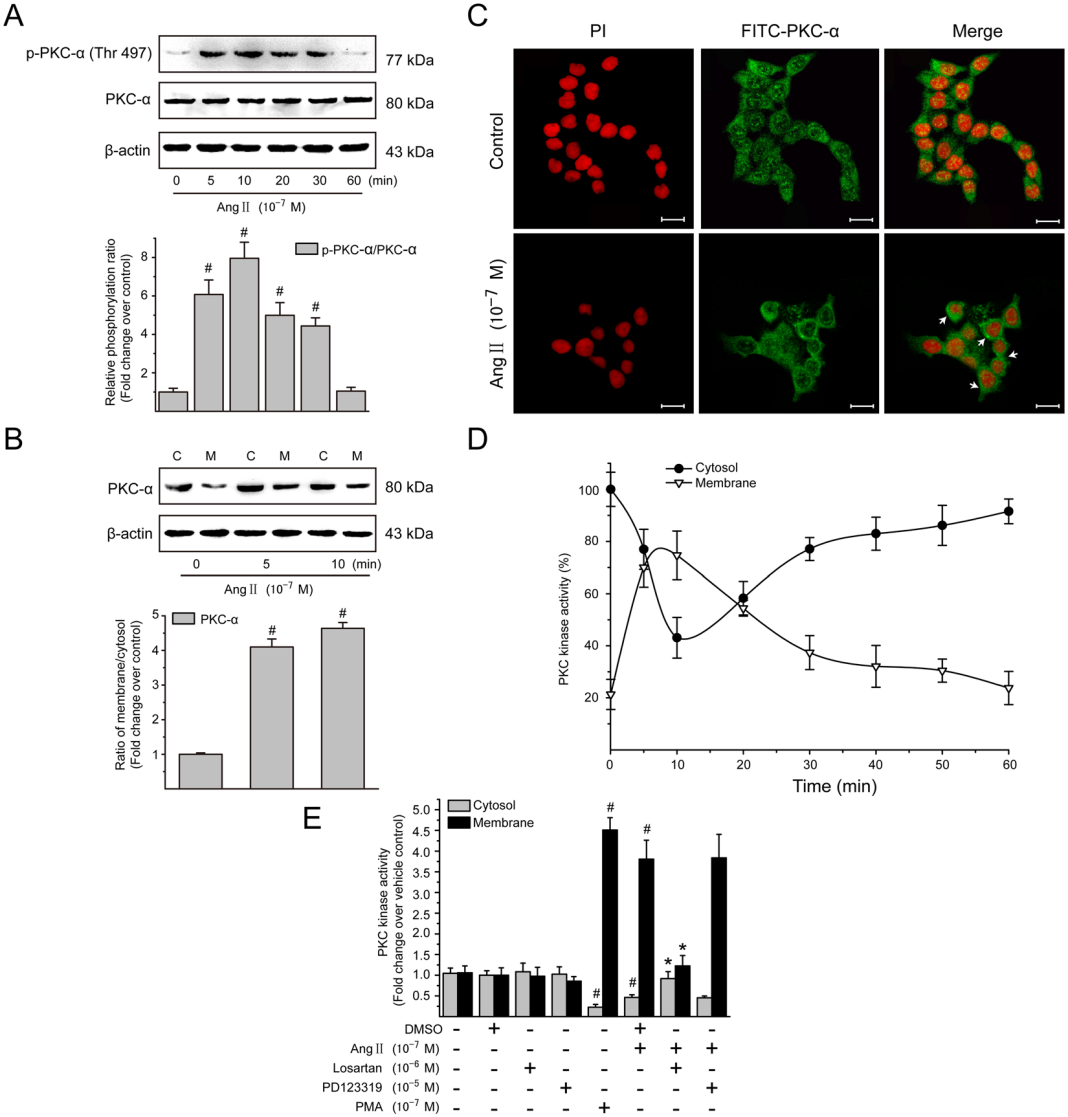
Ang II induces PKC-*α* activation *via* AT_1_ in LX-2 cells. (**A**) Serum-starved LX-2 cells were exposed to Ang II (10^−7^ M) for 0, 5, 10, 20, 30 and 60 min. Whole cell protein extracts were then subjected to immunoblotting analysis. Similar results were observed in 3 independent experiments, and representative immunoblot bands probed with antibodies against phospho-specific and total PKC-*α* are shown. Total protein level of PKC-*α* served as an internal control. (**B**) Serum-starved LX-2 cells were stimulated with Ang II (10^−7^ M) for 0, 5 and 10 min. Cytoplamic (**C**) and membraneous (M) fractions were analyzed by immunoblotting with an antibody against PKC-*α*. Similar results were observed in 3 independent experiments, and a representative immunoblot band of PKC-*α* is shown. The membrane-to-cytosol ratio was used to calculate fold translocation (or activation) over that of unstimulated cells. (**C**) Serum-starved LX-2 cells were treated with Ang II (10^−7^ M) for 10 min, and incubated with primary antibody against PKC-*α*, followed by incubation with FITC-conjugated IgG (green). The nuclei were revealed with PI (red). Cytosol to cell membrane translocation of PKC-*α* was visualized and photographed by a confocal laser scanning microscope. Scale bar, 20 μm. (**D**) Serum-starved LX-2 cells were stimulated with Ang II (10^−7^ M) for 0–60 min. Graphical presentation showing time course of the relative kinase activity of PKC in the cytosolic and membrane fractions. The PKC kinase activity was expressed as the percentage of the cytosolic value in unstimulated control (100%). (E) Serum-starved LX-2 cells were preincubated with losartan (10^−6^ M) or PD123319 (10^−5^ M) for 1 h and then stimulated with Ang II (10^−7^ M) for 10 min. PMA (10^−7^ M), a PKC activator, was used as a positive control. PKC enzyme activities in nuclear and membranous protein lysates were quantified using a colorimetric PKC Kinase Activity Assay Kit. The PKC activity in vehicle control cells was set as 1. Data are presented as mean ± SD of 3 independent experiments. ^#^ *P* < 0.05 *versus* unstimulated or vehicle-treated control cells; ^*^ *P* < 0.05 *versus* Ang II-treated cells.

It is well established that the PKC activity controls Ang II-stimulated cellular events^25^. PKC-*α* was the classic PKC isoform whose phosphorylated form in LX-2 cells was rapidly induced in less than 5 min (Fig. 3A) after the addition of Ang II (10^−7^ M), reaching a peak level at 10 min and then returning to a basal level after 1 h of stimulation. In addition to phosphorylation, PKC-*α* redistribution from cytoplasm to cell membrane reflects intracellular PKC-*α* activation^26^. Thus, levels of PKC-*α* protein in the cytosolic and membrane fractions were subsequently evaluated by immunoblotting analysis. Treatment with Ang II (10^−7^ M) caused a rapid translocation of PKC-*α* to the membrane fraction accompanied with a marked decrease in PKC-*α* that appeared in the cytosol (Fig. 3B). The membrane-to-cytosol ratios of PKC-*α* protein after 5 and 10 min of Ang II treatment were 4.10- and 4.64-fold higher than the initial baseline value in the absence of Ang II. Immunofluorescence staining also revealed subcellular distribution of PKC-*α* in LX-2 cells. PKC-*α* was found mainly in cytoplasmic compartment of untreated LX-2 cells (Fig. 3C). After a 10 min treatment with Ang II, PKC-*α* showed a cell membrane distribution. Consistent with cell translocation of PKC-*α*, Ang II (10^−7^ M) stimulation resulted in a rapid and transient reduction in cytosolic PKC activity (Fig. 3D) within 10 min in cultured LX-2 cells. Afterwards, the PKC activity in the cytosolic fraction gradually increased with the duration of treatment, but still remained less than that in control group until 1 h after stimulation. In contrast, after stimulation with Ang II, the PKC activity in the membrane fraction increased rapidly with a maximal peak at 10 min later, and it then decreased gradually. In addition, Ang II-mediated effects on PKC activity were completely abolished by pretreatment with losartan in LX-2 cells (Fig. 3E), signified by a simultaneous decrease in cytoplasmic PKC activity and increase in cell membrane. However, PD123319 had no effect on Ang II-induced changes in cytosolic and membrane-associated PKC activities. Taken together, these data strongly suggest that Ang II-induced PKC activation in LX-2 cells is mediated by AT_1_, rather than by AT_2_.

### PKC activity is required for Ang II-mediated MAPK signaling in LX-2 cells

**Figure 4:**
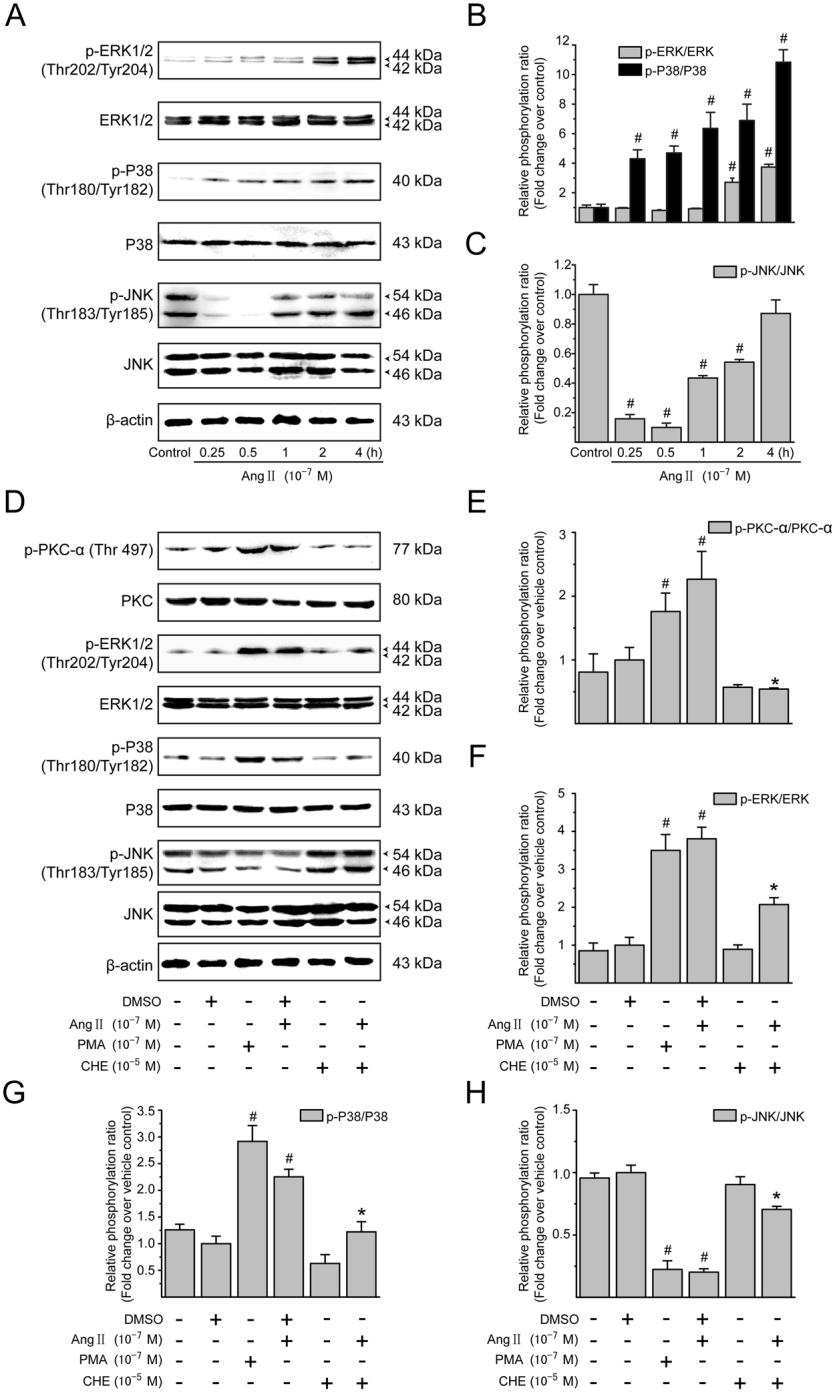
Ang II-mediated MAPK pathways depend on PKC activation in the LX-2 cells. (**A**) Serum-starved LX-2 cells were incubated with Ang II (10^−7^ M) for 0, 0.25, 0.5, 1, 2 and 4 h. Whole cell lysates were immunoblotted with antibodies against ERK1/2, p38 MAPK, JNK, and their phosphorylated forms, respectively. Total protein levels of ERK1/2, p38 MAPK and JNK were used as internal controls. Similar results were observed in 3 independent experiments, and representative immunoblot bands are shown. (**B,C**) Fold-changes in the relative levels of phosphorylated ERK1/2, p38 MAPK and JNK protein are shown after normalizing with the corresponding loading control. The relative protein level in vehicle-treated cells was set as 1.0. (**D**) Serum-starved LX-2 cells were pretreated with CHE (a PKC inhibitor; 10^−5^ M) for 0.5 h, and then incubated with or without Ang II (10^−7^ M) for either 0.5 h (to detect phosphorylated PKC-*α*, p38 MAPK and JNK) or 2 h (to detect phosphorylated ERK1/2), followed by Western blotting with the indicated antibodies. PMA (10^−7^ M) was included as a positive control. Total protein levels of PKC-*α*, ERK1/2, p38 MAPK and JNK were used as loading controls. Similar results were observed in 3 independent experiments, and representative immunoblots for each protein are shown. (**E–H**) The protein levels of phosphorylated forms of PKC-*α*, ERK1/2, p38 MAPK and JNK were normalized with the value of respective loading control. It was designated as relative phosphorylation ratio compared with that in vehicle-treated cells (defined as 1-fold). Results are presented as mean ± SD of 3 independent experiments. ^#^ *P* < 0.05 *versus* unstimulated or vehicle-treated control cells; ^*^ *P* < 0.05 *versus* Ang II-treated cells.

A potential downstream target for PKC-mediated signaling pathways is the family of MAPKs, which are activated through the phosphorylation on their key tyrosine and threonine residues^18^. Figure 4A–C show the effect of Ang II (10^−7^ M) on the phosphorylation and activation of the 3 MAPKs (ERK1/2, p38 MAPK, and JNK) over a 4-h incubation period in LX-2 cells by employing antibodies specific to the phosphorylated forms of MAPKs. In Ang II-treated cultures of LX-2 cells, the level of ERK1/2 phosphorylation was delayed in the first 1 h but strongly increased between 2 and 4 h. Ang II also caused a rapid phosphorylation of p38 MAPK within 15 min, and this increase remained significant through the longest incubation of 4 h. However, Ang II induced a transient dephosphorylation and inactivation of JNK. The phosphorylation level of JNK declined to a nadir at 0.5 h and then returned to basal level after a 4-h incubation with Ang II.

To further evaluate whether the MAPKs are regulated by PKC, the effects of CHE, a broad-spectrum PKC inhibitor, on the phosphorylation state of PKC-*α* as well as each MAPK were examined. Treatment of cells with CHE did not alter the basal state of PKC-*α* and MAPK phosphorylation (Fig. 4D–G), but effectively suppressed Ang II-induced phosphorylation of PKC-*α* (Fig. 4D,E), ERK1/2 (Fig. 4D,F) and p38 MAPK (Fig. 4D,G), and eliminated the inhibitory effect of Ang II on JNK phosphorylation (Fig. 4D,H). Furthermore, it was also noted that the classical PKC activator PMA, as a positive control, had similar effects as Ang II on MAPKs phosphorylation/dephosphorylation. These results suggest that Ang II-mediated MAPK pathways are dependent on the PKC activation in LX-2 cells.

### Ang II mediates NF-*κ*B activation in LX-2 cells

**Figure 5:**
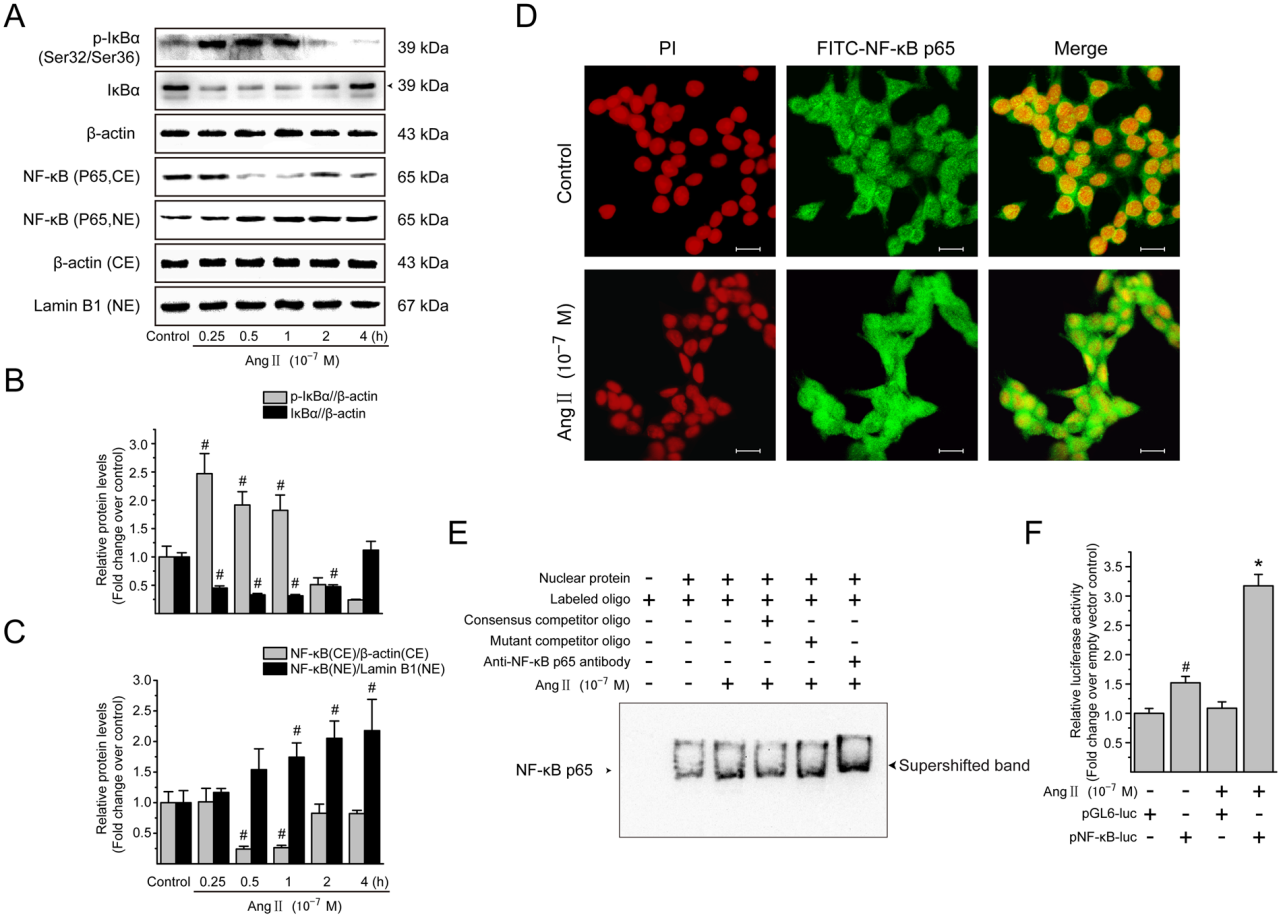
Ang II activates the NF-*κ*B pathway in LX-2 cells. (**A**) Serum-starved LX-2 cells were treated with Ang II (10^−7^ M) for 0, 0.25, 0.5, 1, 2 and 4 h. Whole cell lysates were immunoblotted with antibodies against I*κ*B*α* and its phosphorylated form. *β*-Actin was used as an internal control. Cytosolic and nuclear extracts were analyzed by immunoblotting for detection of NF-*κ*B p65. The *β*-actin and lamin B1 protein levels served as internal controls, respectively, for cytoplasmic extracts (CE) and nuclear extracts (NE). (**B**) The protein levels of phosphorylated I*κ*B*α* and I*κ*B*α* in whole cell lysates, and (**C**) that of NF-*κ*B p65 in cytoplasmic and nuclear extracts, were converted to arbitrary densitometric units, normalized by the value of the corresponding internal control and expressed according to the levels in unstimulated cells (defined as 1-fold). (**D**) Immunofluorescence staining was carried out with primary NF-*κ*B p65 antibody followed by incubation of a FITC-labeled secondary antibody (green). The nuclei were stained with PI (red). In the merged micrographs, the yellow color indicates nuclear localization signal of NF-*κ*B p65 subunit. Scale bar, 20 μm. (**E**) Serum-starved LX-2 cells were stimulated in the absence or presence of Ang II (10^−7^ M) for 0.5 h. Nuclear extracts were prepared and the binding activity of NF-*κ*B with the consensus binding sequences was analyzed by EMSA, as described in Materials and Methods. The DNA-protein interaction was resolved on a 6% native polyacrylamide gel and the biotin-labeled DNA bands were visualized by an ECL assay Kit. (**F**) Serum-starved LX-2 cells were transfected with either firefly luciferase reporter of NF-*κ*B (pNF-*κ*B-luc) or pGL6-luc reporter construct followed by incubation with Ang II (10^−7^ M) for 0.5 h. The *Renilla* luciferase reporter was also co-transfected into the cells for normalizing transfection efficiency. The luciferase activity in the cellular extracts was evaluated by the Dual-Luciferase Reporter Assay System and normalized to the activity of *Renilla*. Experiments were repeated 3 times with similar results, and all data are presented as mean ± SD. ^#^ *P* < 0.05 *versus* unstimulated or pGL6-luc empty vector-transfected control cells; ^*^ *P* < 0.05 *versus* pNF-*κ*B-luc-transfected cells without Ang II stimulation.

Recent studies demonstrated that NF-*κ*B is involved in the regulation of CTGF transcription^24, 27^. Therefore, we first investigated whether Ang II mediates transcriptional activation of NF-*κ*B by phosphorylation and degradation of its inhibitory protein inhibitory kappa B (I*κ*B). As shown in Fig. 5A,B, Ang II (10^−7^ M) rapidly increased the phosphorylation level of I*κ*B*α* at 15 min after administration, and the increase was maintained over a 1-h experimental period; meanwhile, Ang II also induced rapid and sustained degradation of I*κ*B*α* from 15 min to 2 h post-treatment.

Translocation of NF-*κ*B from cytoplasm to nucleus is required for its transcriptional activity^28^. Thus, the relative levels of NF-*κ*B p65 subunit in the cytoplasm and nucleus were examined by immunoblotting analysis (Fig. 5A,C). Ang II treatment for 0.5 h induced NF-*κ*B p65 subunit translocation into nuclei of LX-2 cells, demonstrated by a reduced cytoplasmic level but an increased nuclear level, which was also confirmed by immunofluorescence staining for NF-*κ*B p65 subunit (Fig. 5D).

Except for evaluation of protein expression of phosphorylated and total I*κ*B, as well as nuclear translocation of NF-*κ*B, we also focused on the DNA binding and transcriptional regulatory activities of NF-*κ*B, based on electrophoretic mobility shift assays (EMSA) and luciferase reporter activity assay, respectively. Treatment of LX-2 cells with Ang II led to a significant increase in the binding activity of biotin-labeled exogenous consensus DNA oligonucleotides with NF-*κ*B in nuclear fraction (Fig. 5E, lane 3). The density of the complex band was apparently reduced by the addition of unlabeled wild-type NF-*κ*B probes (Fig. 5E, lane 4), whereas it was not altered by the addition of unlabeled mutant probes (Fig. 5E, lane 5). Supershifted band (Fig. 5E, lane 6) that results from the binding of specific NF-*κ*B p65 antibody also confirmed Ang II induced the formation of DNA-NF-*κ*B complex in the nucleus. In addition, the results of dual-luciferase reporter assay with pNF-*κ*B-Luc, which includes multiple NF-*κ*B response elements, showed a higher transcriptional regulatory activity of NF-*κ*B was induced by Ang II treatment than that in pNF-*κ*B-Luc-transfected control cells (Fig. 5F). Collectively, these results show that Ang II induces NF-*κ*B activation in LX-2 cells.

### Ang II activates Smad pathway in LX-2 cells

**Figure 6:**
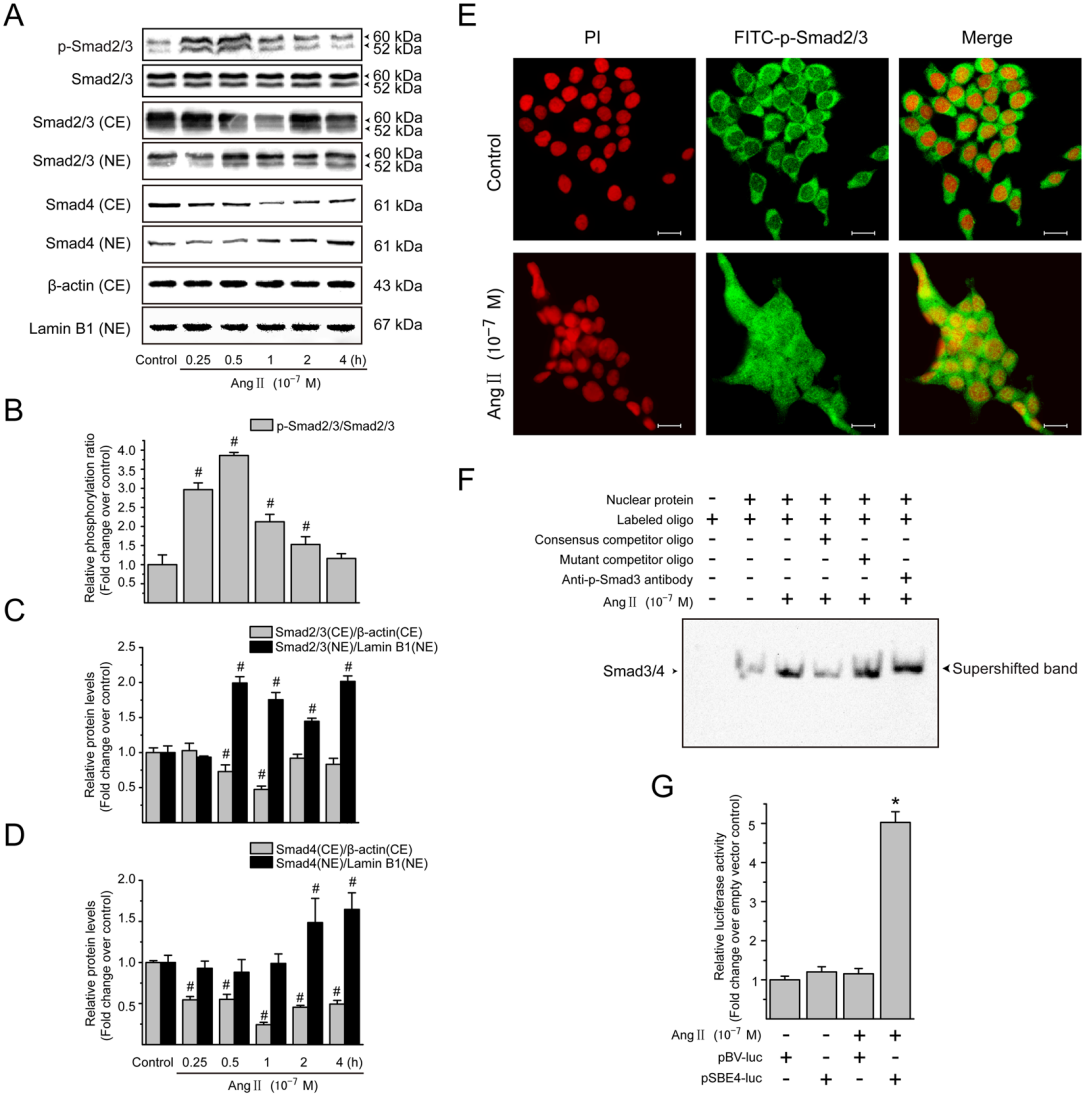
Ang II induces the Smad activation in LX-2 cells. (**A**) Serum-starved LX-2 cells were incubated with Ang II (10^−7^ M) for 0, 0.25, 0.5, 1, 2 and 4 h. Whole cell lysates were subjected to immunoblotting analysis using phosphorylated Smad2/3 antibodies, and total protein levels of Smad2/3 were used as internal controls. Cytoplasmic and nuclear fractions were prepared and analyzed by Western blotting to detect Smad2/3 and Smad4. The *β*-actin and lamin B1 protein levels were used as internal controls, respectively, for cytoplasmic extracts (CE) and nuclear extracts (NE). (**B–D**) The protein levels of phosphorylated Smad2/3 in whole cell extracts, and Smad2/3 and Smad4 in cytoplasmic and nuclear extracts were converted to arbitrary densitometric units, normalized by the value of the corresponding loading control and expressed relative to the phosphorylation ratio or the protein level as *n*-fold over unstimulated cells (defined as 1-fold). (**E**) The localization of phosphorylated Smad2/3 was examined by immunofluorescence staining following treatment of LX-2 cells with Ang II (10^−7^ M) for 0.5 h. The fluorescence images were observed and captured by a confocal laser scanning microscope. Scale bar, 20 μm. (**F**) Serum-starved LX-2 cells were incubated with Ang II (10^−7^ M) for 0.5 h. Nuclear proteins were extracted and the binding activity of Smad3/4 with the consensus binding sequences was analyzed by EMSA, as described in Materials and Methods. The binding mixtures were separated on a 6% nondenaturing polyacrylamide and the DNA-protein complexes were visualized with an ECL assay Kit. (**G**) Serum-starved LX-2 were transfected with either Smad binding element-luciferase reporter plasmid (pSBE4-luc) or pBV-Luc reporter construct, and then stimulated by Ang II (10^−7^ M) for 0.5 h. The *Renilla* luciferase reporter plasmid was used as the internal control of transfection efficiency. The firefly or *Renilla* luciferase activity in total cell extracts was determined using the Dual-Luciferase Reporter Assay System. Experiments were repeated 3 times with similar results, and all data are presented as mean ± SD. ^#^ *P* < 0.05 *versus* unstimulated control cells; ^*^ *P* < 0.05 *versus* pSBE4-luc-transfected cells without Ang II stimulation.

A critical step in the activation of Smad pathway is the phosphorylation of receptor-associated Smads (R-Smads; Smad2/3)^29^. As illustrated in Fig. 6A,B, stimulation with Ang II (10^−7^ M) induced a rapid Smad2/3 phosphorylation in a time-dependent manner in LX-2 cells, being evident at 15 min, peaking at 0.5 h, and declining to baseline level by 4 h. However, the total Smad2/3 protein abundance remained unchanged at different time points observed. Upon phosphorylation and activation by the upstream kinases, Smad2 and Smad3 homo-oligomerize or hetero-oligomerize with Co-Smad Smad4, and translocate to nucleus. Under resting conditions, Smad2/3 was present predominantly in cytoplasmic fraction with only small amount in the nuclear fraction (Fig. 6A,C). Treatment with Ang II for 0.5–1 h resulted in a dramatic decrease in cytoplasmic Smad2/3 protein levels and great increase of nuclear Smad2/3 protein levels. Consistent with these observations, Ang II caused a marked nuclear translocation of Smad4 in a time-dependent manner (Fig. 6A,D). The Ang II effect on Smad2/3 activation was also certified by immunofluorescence staining of phosphorylated Smad2/3. In growth-arrested LX-2 cells, phosphorylated Smad2/3 proteins were located almost exclusively in the cytosol (Fig. 6E); Ang II treatment led to the nuclear translocation of phosphorylated Smad2/3.

In the nucleus, transcription of target genes is activated through the binding of the R-Smads–Smad4 to specific DNA sequences^30^. Corresponding to a time point with maximal induction of Smad translocation, Ang II stimulation for 0.5 h increased the DNA-binding activity of R-Smads–Smad4 complex in nuclear extracts (Fig. 6F, lane 3). The specificity of binding was detected by competition assays with excess amount of unlabeled (Fig. 6F, lane 4) or mutant (Fig. 6F, lane 5) Smad3/4 probes. Phosphorylated Smad3 antibody was also used to supershift the DNA–R-Smads–Smad4 complex to confirm specific binding (Fig. 6F, lane 6). By transient transfection with a luciferase reporter plasmid (pSBE4-Luc) consisting of four Smad binding elements (SBE), we found that Ang II had a positive effect on Smad transcriptional activity in LX-2 cells (Fig. 6G). Taken together, these results suggest that Ang II induces the transcriptional activation of Smad complexes in LX-2 cells.

### Effect of PKC, MEK1/2, or p38 MAPK inhibitor on Ang II-mediated NF-*κ*B and Smad activation in LX-2 cells

**Figure 7:**
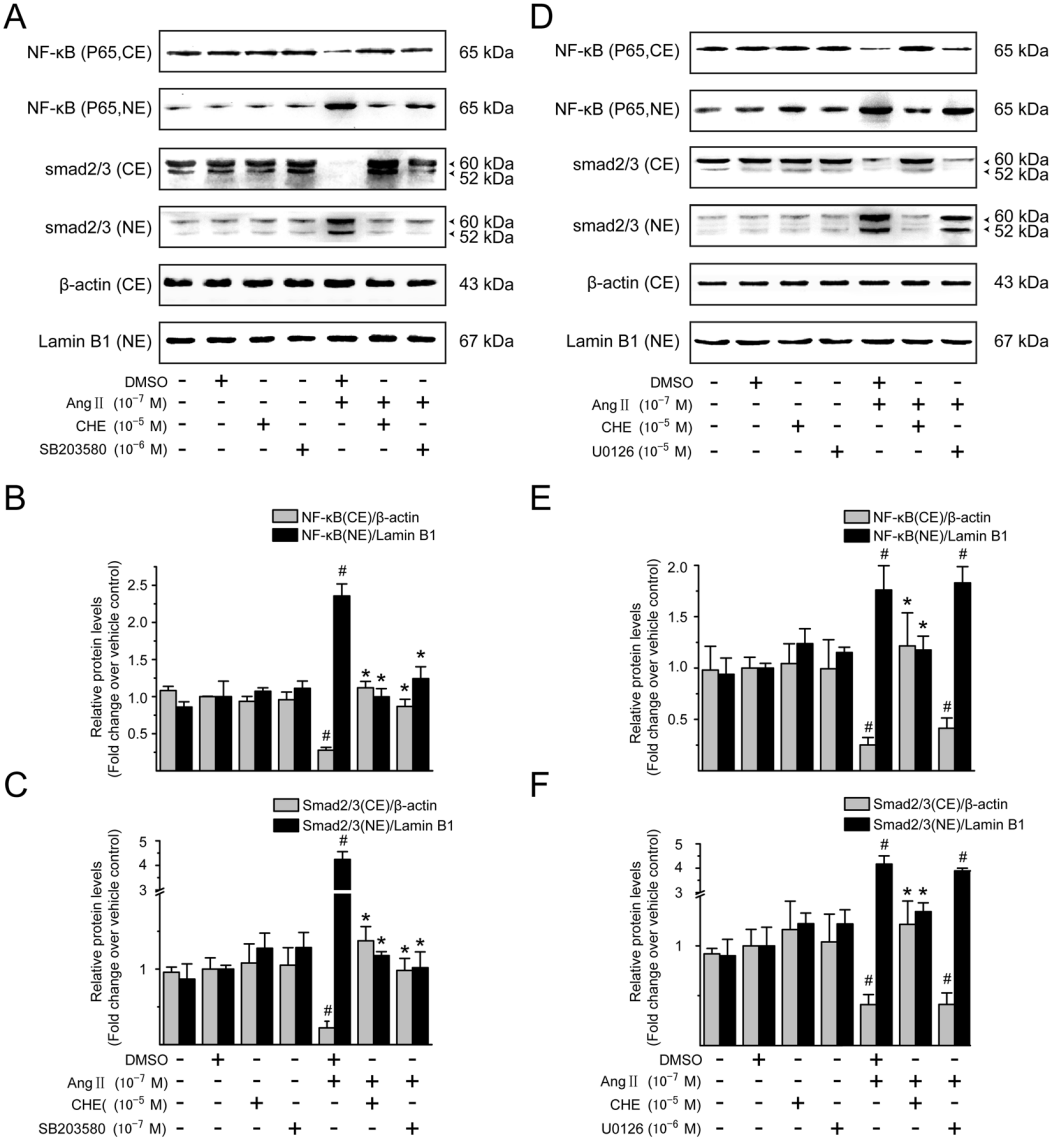
PKC/p38 MAPK, but not ERK1/2 MAPK signaling, mediates Ang II-induced Smad activation. Serum-starved LX-2 cells were preincubated for 0.5 h with CHE (a PKC inhibitor; 10^−5^ M), SB203580 (a p38 MAPK inhibitor; 10^−5^ M), or U0126 (an inhibitor of MEK1/2, upstream of ERK1/2 MAPK; 10^−6^ M) in the presence and absence of Ang II (10^−7^ M) stimulation for 0.5 h. Cytoplasmic extracts (CE) and nuclear extracts (NE) were prepared and then analyzed by immunoblotting with antibodies against NF-*κ*B p65 and Smad2/3. *β*-Actin and lamin B1 were used as the internal control for the CE and NE, respectively. (**A,D**) Representative immunoblot bands for NF-*κ*B p65 and Smad2/3 are shown. Results of (**B,E**) NF-*κ*B p65 and (**C,F**) Smad2/3 levels were obtained by densitometric analysis, normalized by the value of the corresponding internal control and expressed relative to the protein level in vehicle control cells (defined as 1-fold). Experiments were repeated 3 times with similar results, and data are presented as mean ± SD. ^#^ *P* < 0.05 *versus* vehicle-treated control cells; ^*^ *P* < 0.05 *versus* Ang II-treated cells.

To clarify whether the PKC-mediated ERK1/2 and p38 MAPK pathways are responsible for Ang II-induced NF-*κ*B and Smad activation, we first examined the effects of CHE, the PKC inhibitor, on Ang II-induced activation and nuclear translocation of NF-*κ*B and Smad2/3. Pretreatment with CHE caused a simultaneous decrease in nucleic NF-*κ*B p65 and increase in cytoplasm in Ang II-stimulated LX-2 cells (Fig. 7A,B,D,E). Similarly, Ang II-induced Smad2/3 nuclear accumulation was also effectively prevented by pretreatment with CHE (Fig. 7A,C,D,F), indicating that the activation of NF-*κ*B and Smad2/3 pathways occurs in a PKC dependent manner.

We next sought to test whether ERK1/2 or p38 MAPK pathway is involved in Ang II-mediated NF-*κ*B and Smad activation. Pretreatment of LX-2 cells with p38 MAPK inhibitor SB203580 significantly blocked Ang II-induced nuclear translocation of NF-*κ*B and Smad2/3 (Fig. 7A–C), while no effect was observed in cells treated by U0126, a specific MEK1/2 (MAPK/ERK kinase) inhibitor (Fig. 7D–F). In addition, these pharmacological inhibitors alone had little effect on the NF-*κ*B and Smad pathway activation, and therefore nucleic localization. These results show the Ang II-induced NF-*κ*B and Smad activation is mediated by p38 MAPK but not by ERK1/2 MAPK signaling in LX-2 cells.

### Role of JNK dephosphorylation in Ang II-mediated NF-*κ*B and Smad activation in LX-2 cells

**Figure 8:**
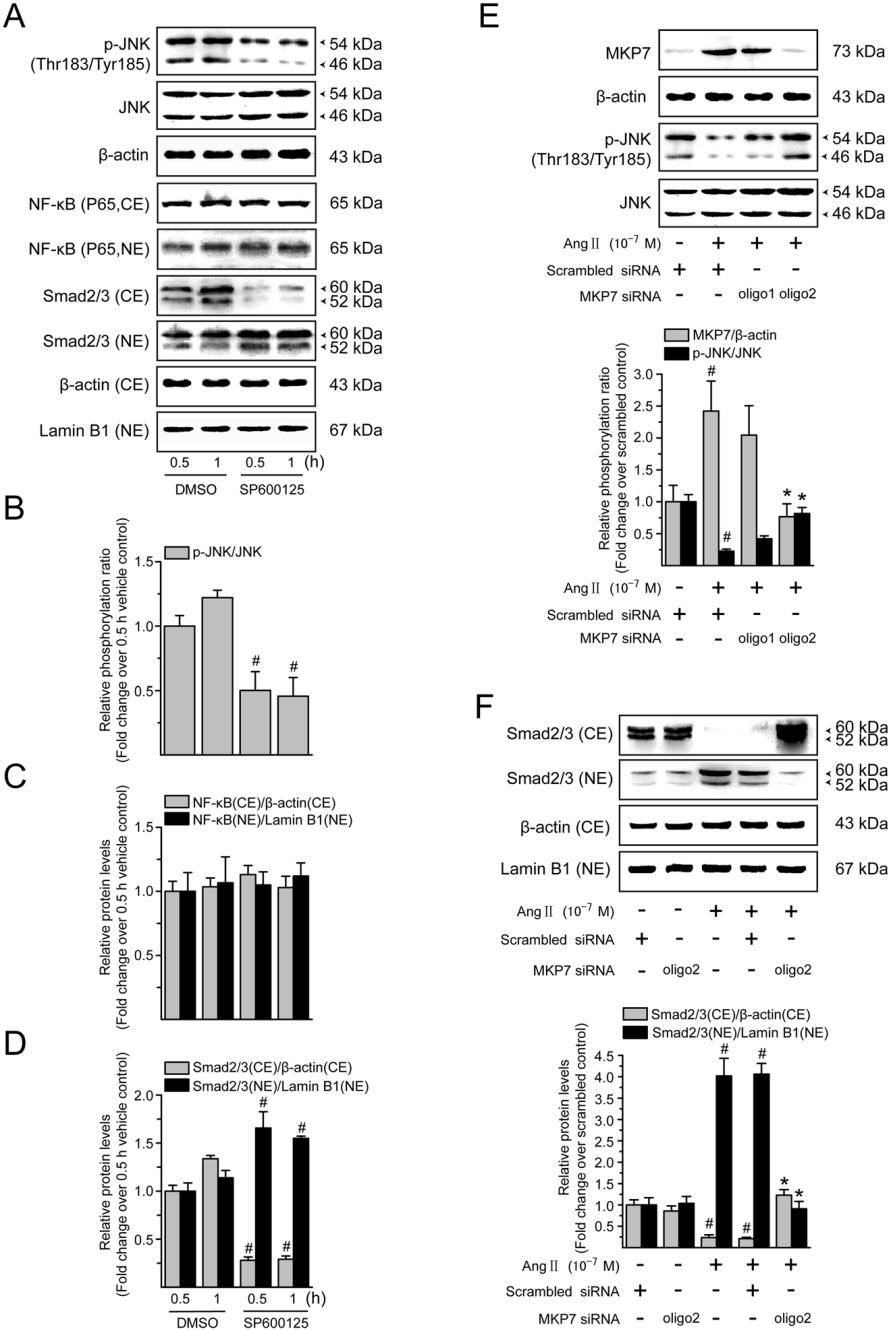
Ang II-mediated Smad activation involves JNK dephosphorylation in LX-2 cells. (**A**) Serum-starved LX-2 cells were incubated with SP600125 (a JNK inhibitor; 10^−5^ M) for 0.5 and 1 h. Whole cell extracts were immunoblotted with antibodies against JNK and its phosphorylated form. Total protein level of JNK in whole cell extracts was used as an internal control. Cytoplasmic extracts (CE) and nuclear extracts (NE) were subjected to immunoblotting with antibodies against NF-*κ*B p65 and Smad2/3. *β*-Actin and lamin B1 protein levels were used as internal controls, respectively, for the CE and NE. (**B**) Levels of phosphorylated JNK in whole cell lysates, and (**C**) that of NF-*κ*B p65 and (**D**) Smad2/3 in cytoplasmic and nuclear extracts, were converted to arbitrary densitometric units, normalized to the corresponding internal control and expressed relative to the phosphorylation ratio or to the protein level in cells treated with vehicle for 0.5 h (defined as 1-fold). (**E**) Serum-starved LX-2 cells were transiently transfected with scrambled or MKP7 siRNA. After transfection for 48 h, cells were incubated with serum free medium containing Ang II (10^−7^ M) for a further 0.5 h. To ensure the adequate knockdown of MKP7 and inhibition of JNK dephosphorylation, whole cell lysates were subjected to immunoblotting analysis with antibodies against MKP7 and phosphorylated JNK. The expression of *β*-actin and total JNK was monitored to confirm equal protein loading. (**F**) Cytoplasmic extracts (CE) and nuclear extracts (NE) were prepared and levels of Smad2/3 were evaluated by Western blotting. *β*-Actin and lamin B1 protein signals served as controls for protein loading of the CE and NE, respectively. Results of Smad2/3 protein expression in cytoplasmic and nuclear fractions were obtained from densitometric analysis, normalized by corresponding internal control and expressed relative to the protein level in scrambled siRNA-treated control cells (defined as 1-fold). All data are shown as mean ± SD of 3 independent experiments. ^#^ *P* < 0.05 *versus* vehicle- or scrambled siRNA-treated control cells; ^*^ *P* < 0.05 *versus* Ang II + scrambled siRNA-treated cells.

Since the change of JNK dephosphorylation was observed in Ang II-stimulated LX-2 cells, the specific JNK pathway inhibitor, SP600125, was employed to further investigate the role of the JNK in the Ang II-induced NF-*κ*B and Smad activation. Similar to Ang II, SP600125 could effectively suppress the JNK phosphorylation (Fig. 8A,B) and induce Smad translocation from the cytoplasm into the nucleus (Fig. 8A,D). However, in our experiments, SP600125 did not alter nuclear translocation of NF-*κ*B detected at 0.5 or 1 h of incubation with Ang II (Fig. 8A,C), suggesting that Ang II-mediated JNK dephosphorylation is not involved in NF-*κ*B activation pathway in LX-2 cells.

To further investigate whether Ang II-induced JNK dephosphorylation is required for Smad activation, we use the small interfering RNA (siRNA) approach to specifically knockdown MKP7, a JNK-specific phosphatase. As shown in Fig. 8E, MKP-7 protein expression was elevated in scrambled siRNA-expressing cells following Ang II treatment, whereas MKP-7 induction was largely eliminated by the MKP-7 siRNA duplexes. Meanwhile, the effect of Ang II on JNK dephosphorylation was significantly attenuated in cells treated with transient transfection of No. 2 siRNA duplexes of MKP7, which specifically suppressed Ang II-induced MKP7 expression by approximately 68%, compared with cells transfected with scrambled siRNA followed by Ang II stimulation. In a quiescent condition, the inhibition by siRNA-mediated knockdown of MKP7 had no obvious effect on the Smad2/3 expression in the cytoplasmic and nuclear fractions of LX-2 cells (Fig. 8F), while that by MKP7 siRNA suppressed Ang II-induced Smad2/3 nuclear translocation, showing higher cytoplasmic and lower nuclear Smad2/3 protein levels compared with those in the scrambled siRNA transfected cells. These results suggest that Ang II-induced Smad activation may be mediated, at least in part, *via* the JNK dephosphorylation and therefore inactivation in LX-2 cells.

### Ang II-induced CTGF and ECM protein expression is mediated by NF-*κ*B and Smad activation in LX-2 cells

**Figure 9:**
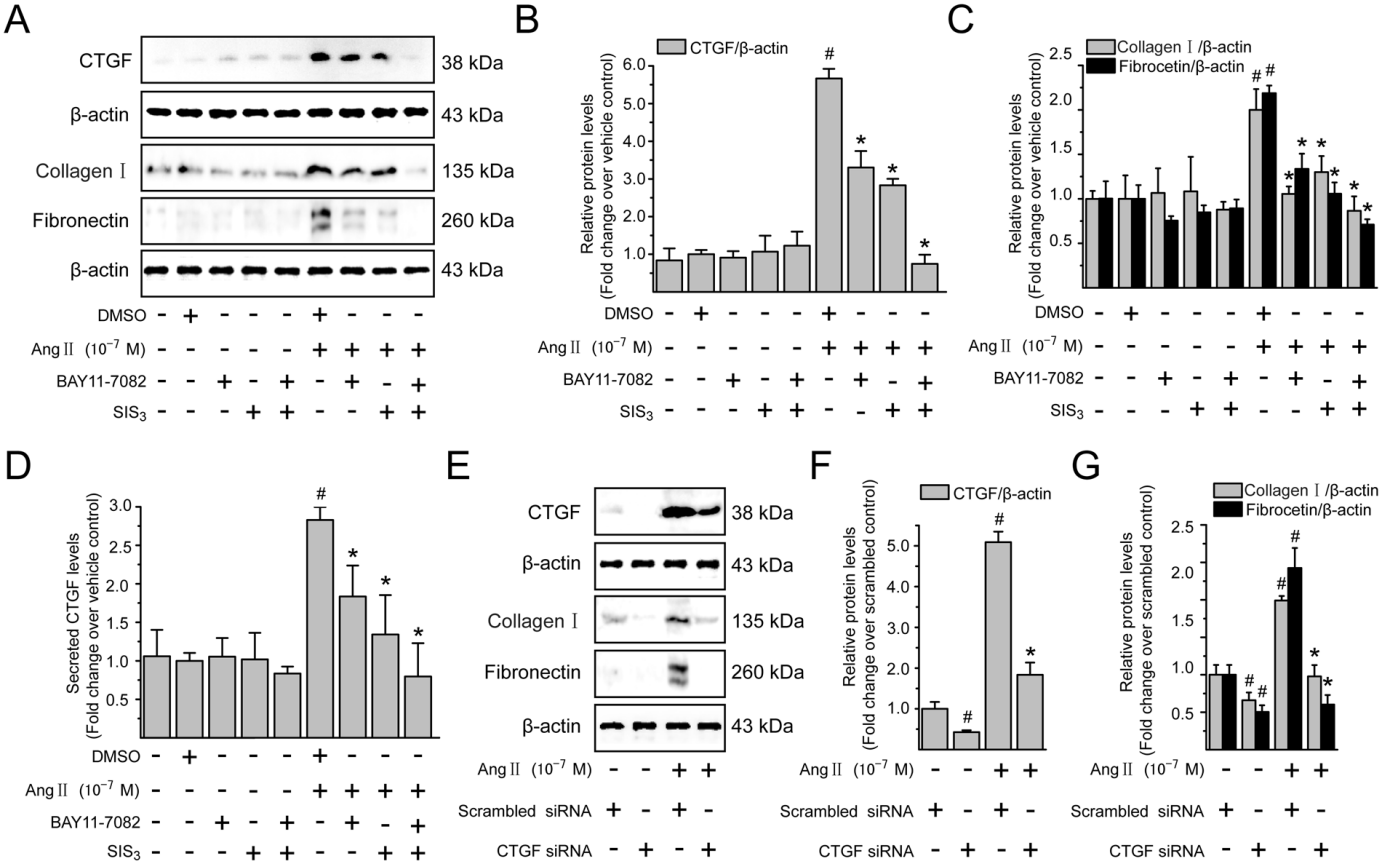
Ang II *via* NF-*κ*B and Smad pathways increases CTGF production and CTGF is involved in Ang II-induced ECM accumulation in LX-2 cells. Serum-starved LX-2 cells were preincubated for 0.5 h with BAY11-7082 (a specific NF-*κ*B inhibitor; 10^−6^ M), or SIS3 (a specific Smad3 inhibitor, 10^−6^ M) alone or simultaneously in the presence or absence of Ang II (10^−7^ M) stimulation for 4 h (to detect CTGF) or 24 h (to detect type I collagen and fibronectin). Whole cell lysates were immunoblotted with antibodies against CTGF, type I collagen and fibronectin, respectively. *β*-Actin level served as a control for equal protein loading. (**A**) Representative immunoblot bands are shown for the indicated antibodies. (**B,C**) The histogram represents results of the densitometric scans for the protein bands of CTGF, type I collagen and fibronectin after normalization with *β*-actin. (**D**) CTGF concentrations in the cell supernatants were determined by ELISA, and the value in the vehicle control group was defined as 1.0. (**E**) Serum-starved LX-2 cells transiently transfected with scrambled siRNA or CTGF siRNA were treated with Ang II (10^−7^ M) for 4 h (to detect CTGF) or 24 h (to detect type I collagen and fibronectin), respectively. After treatment, aliquots of whole cell lysates were subjected to immunoblotting with specific antibodies as indicated. *β*-Actin was used as an internal control. (**E**) The experiments were repeated thrice with similar results and representative immunoblot bands for CTGF, type I collagen and fibronectin are shown. (**F,G**) Fold-change in relative protein level of each protein is shown after normalizing with *β*-actin. All data are presented as mean ± SD of 3 independent experiments. ^#^ *P* < 0.05 *versus* vehicle- or scrambled siRNA-treated control cells; ^*^ *P* < 0.05 *versus* Ang II- or Ang II + scrambled siRNA-treated cells.

We next evaluated the involvement of NF-*κ*B and Smad activation in Ang II-induced CTGF expression. To block NF-*κ*B and Smad actions, before stimulation with Ang II (10^−7^ M), LX-2 cells were pretreated with BAY11-7082 or SIS3, which specifically inhibited the I*κ*B*α* and Smad3 phosphorylation, respectively. The protein expression (Fig. 9A,B) and secretion (Fig. 9D) of CTGF induced by Ang II were modestly diminished by BAY11-7082 or SIS3 alone, and BAY11-7082 in combination with SIS3 produced an additive effect in suppressing the Ang II-induced CTGF production and secretion. However, no effect was seen on control cells with these inhibitors, no matter administered alone or in combination. Similarly, NF-*κ*B and/or Smad signaling inhibitors markedly reduced the enhancement effect of Ang II on expression of ECM components, including type I collagen and fibronectin (Fig. 9A,C). These data suggest that the raised CTGF expression and accelerated ECM accumulation in response to Ang II stimuli require the participation of NF-*κ*B and Smad signaling in LX-2 cells.

### Ang II-induced ECM protein accumulation is dependent on CTGF overexpression in LX-2 cells

Several studies have indicated CTGF as a mediator underlying the fibrotic remodeling process^15, 16^. However, it remains unclear whether CTGF functions as a direct downstream effector in mediating Ang II-induced fibrogenic responses in HSCs. Therefore, LX-2 cells were transiently transfected with either scrambled or CTGF siRNA, and the knockdown effectiveness was confirmed by immunoblotting analysis (Fig. 9E,F). As shown in Fig. 9E,G, knockdown of CTGF strikingly attenuated Ang II-induced increase in type I collagen and fibronectin protein levels compared with cells transfected with scrambled siRNA. These data suggest that CTGF is a key downstream mediator of the profibrotic effects of Ang II in LX-2 cells.

## Discussion

The main finding of the current study was that Ang II could induce a rapid expression of CTGF in human HSCs directly *via* the AT_1_ receptor-mediated PKC*α* activation, which driven an early p38 MAPK-mediated Smad and NF-*κ*B cross-talk pathway, in addition to a late TGF-*β*-dependent mechanism. Interestingly, we also provided new evidence that dephosphorylation but not phosphorylation of JNK was a key signaling mechanism required for the upregulation of CTGF expression in response to Ang II.

Accumulating evidence indicates that upregulation of CTGF might represent a pivotal pathway during HSC activation and hepatic fibrosis, because it plays a central role as a mitogen, fibroblast chemoattractant, and inducer of ECM component synthesis and secretion in fibrotic liver^6^. CTGF is mainly produced by activated HSCs during the process of liver fibrogenesis, and blockade of CTGF synthesis leads to substantial attenuation of HSC activation and collagen deposition *in vitro* and *in vivo* ^8^. Considerable evidence demonstrates that CTGF functions downstream of TGF-*β*. Many of the fibroblast activating and ECM-forming activities of TGF-*β* are probably mediated by CTGF. Besides the TGF-*β*-dependent signaling pathway, recent studies have also shown that Ang II mediates CTGF and type I collagen overexpression in vascular smooth muscle cells *via* an AT_1_ receptor-mediated ERK/p38 MAPK-Smad cross-talk pathway, independently of TGF-*β* ^22^. Here we have documented that early Ang II-induced CTGF increase in HSCs is TGF-*β* independent but AT_1_ dependent, as indicated by the following 2 findings. First, Ang II upregulated CTGF mRNA and protein expression at as early as 0.5 h, peaking at 1 and 4 h, respectively. The peak CTGF levels preceded the maximum synthesis of TGF-*β*1 protein (observed at 48 h) and could be blocked by losartan (an AT_1_ receptor antagonist). Second, blockade of TGF-*β* signaling could not prevent the early Ang II-dependent CTGF induction because either an inhibitor of TGF-*β* type I receptor kinase activity or a specific siRNA for TGF-*β*1 had no effect on CTGF upregulation at early 4 h. However, late activation of CTGF expression was TGF-*β* mediated, because both TGF-*β* receptor blocker and TGF-*β* siRNA reduced Ang II-induced CTGF protein expression after 24 h incubation.

Ang II had previously been shown to activate PKC isoforms for various cellular responses *in vitro* ^17^. In our model, we observed that Ang II caused both rapid phosphorylation and membrane translocation of PKC*α*, one of the calcium-dependent classic isoforms of PKC. However, after 5 and 10 min of Ang II stimulation, other three non-classical isoforms of PKC^31^, including *δ*, *ε*, and *ζ*, their protein levels in the membranous and cytosolic fractions remained unchanged (Supplementary Fig. S1). These results concur with those previously reported by Kusum^32^, which showed that malonidialdehyde-acetaldehyde-protein adducts utilized the activation of PKC*α* isoform, not the *δ* and *ζ* isoforms, as a possible signaling pathway involved in the PKC membrane translocation and resulting urokinase-type plasminogen secretion in rat HSCs. Since a transient increase in the amount of membrane-associated PKC activity elicited by Ang II coincided with a stimulatory effect on the membrane translocation of PKC-*α*, we speculated that the CTGF induction by Ang II in HSCs was likely mediated by the PKC*α* isoform, even if measurement of other isoforms of PKC was not available.

Sequence analysis of the CTGF promoter has revealed that there are two putative consensus sequences of NF-*κ*B and AP-1, respectively^23^. A putative Smad-binding site (SBE) has also been found to be located at nt −175 to nt −167 in the promoter of *CTGF* gene. Previous works demonstrated that the activation of NF-*κ*B might induce the promoter activity of *CTGF*, leading to the induction of gene expression of *CTGF* in activated HSCs^27^. Similarly, other reports also showed that activation of the NF-*κ*B pathway, independent of the synthesis of TGF-*β*, was required for hypoxia-induced CTGF expression in scleroderma skin fibroblasts^33^. On the other hand, in hepatitis C virus-infected hepatocytes, TGF-*β*1-mediated CTGF overexpression is dependent on the functional Smad element in the *CTGF* promoter^34^. The present study supported that cooperation between NF-*κ*B and Smad might be operative in mediating CTGF induction in response to Ang II stimulation. Interestingly, this type of synergy between NF-*κ*B and Smad for gene regulation is not unprecedented. In the promoter of the pro-survival Bcl2 family member *Mcl-1*, a cooperation between NF-*κ*B and Smad for this gene activation is established, which is critical to TGF-*β*-mediated promotion of osteoclast survival^35^. Therefore, cooperation between NF-*κ*B and Smad could function as a general mechanism to regulate gene transcription. However, basal CTGF expression in rat HSCs is regulated mainly by Ets-1 and Sp1, other two transcription factors having specific binding sites in the promoter region of the *CTGF* gene, but not by Smads^36^. More recently, signal transducer and activator of transcription 3 is implicated as a key mediator in modulating CTGF production upon TGF-*β* treatment in activated HSCs^37^. Therefore, we cannot completely preclude the possibility of other sensitive transcription factors in the regulation of transcriptional activity of *CTGF* promoter. Systematic site-directed mutagenesis and chromosomal immunoprecipitation analysis are being conducted in our team to clearly clarify the transcriptional regulation of *CTGF* gene expression.

Numerous studies have suggested that NF-*κ*B and Smad activation are induced by upstream kinases MAPKs^16, 22, 27^. In the current study, we found that treatment of HSCs with the p38 MAPK inhibitor SB203580 markedly inhibited the nuclear accumulation of NF-*κ*B and Smad2/3, whereas inhibitor of MEK1/2, which operates upstream of ERK1/2, had no effect on the NF-*κ*B and Smad pathways. This is consistent with an earlier report showing that in rat vascular smooth muscle cells, Ang II might signal *via* AT_1_ receptor to activate the Smad signaling pathway; and this mechanism was dependent on the activation of p38 MAPK, but not ERK1/2 signaling^22^. In contrast, previous study has also suggested that the interruption of ERK signaling lead to the reduction in NF-*κ*B activity and in the expression of *CTGF* gene in activated rat HSCs^27^. This discrepancy is currently unclear, but it can be explained partly by the difference in the cell origin of HSCs (i. е., rat *versus* human), but predominantly by the different types of stimulus used (i. е., fetal bovine serum *versus* Ang II). In addition, Ang II-activated Smad signaling was diminished by dominant-negative ERK1/2 and by addition of ERK1/2 inhibitor in mouse primary aorta vascular smooth muscle cells^38^. The contradictory results might be due to the difference of cell types and species or the different intervention strategies to downregulate the ERK1/2 activities.

One of the most interesting findings of our study is that MKP-7 expression was rapidly up-regulated by Ang II, and this induction coincided with the dephosphorylation and inactivation of JNK; and blocking Ang II-induced MKP-7 induction using siRNA increased JNK phosphorylation and repressed nuclear translocation and activation of Smad2/3. MKP-7 is a member of dual specificity phosphatases (DUSPs) family, which inactivates MAPKs by dephosphorylating critical phosphotyrosine and/or phosphothreonine residues. *DUSP* gene expression is induced by lots of cellular stresses and/or growth factors, thereby negatively regulating MAPK superfamily members. MKP-7 binds to and inactivates JNK and p38 MAPK isoforms *α*/*β*, but not ERK^39^. In the present report, we observed that Ang II-induced MKP7 directly dephosphorylated JNK, but not p38 MAPK. This is consistent with previous over-expression studies indicating that MKP7 is more specific for JNK than p38 MAPK^40^. Shima’ group has found activated ERK phosphorylates MKP-7 on a serine residue (Ser-446), thereby stabilizing the protein and blocking JNK activation^41^. This raises an interesting possibility that the late activation of ERK by Ang II may modulate Smad signaling through reducing MKP7 degradation and thereby facilitating JNK inactivation. This hypothesis requires further investigation.

Finally, we investigated whether CTGF was implicated in Ang II-induced profibrotic effects in HSCs. Ang II-induced fibronectin and type I collagen matrix expressions were dependent on the stimulation of CTGF expression in HSCs, as evidenced using a siRNA-based approach to selectively knockdown the *CTGF* gene expression. A similar requirement of CTGF was also observed in the effect of Ang1–7 on changes related to tubular epithelial-mesenchymal transition in rat kidney epithelial cells^42^. Previous data indicate that CTGF is an autocrine effector of Ang II and secreted CTGF may induce its own expression^43^. As a control, we observed that the presence of siRNA CTGF greatly diminished Ang II-induced CTGF overexpression. These data suggest that CTGF acts as a direct downstream mediator of Ang II to stimulate the ECM synthesis in HSCs, which represents an initiating event for hepatic fibrogenesis.

**Figure 10:**
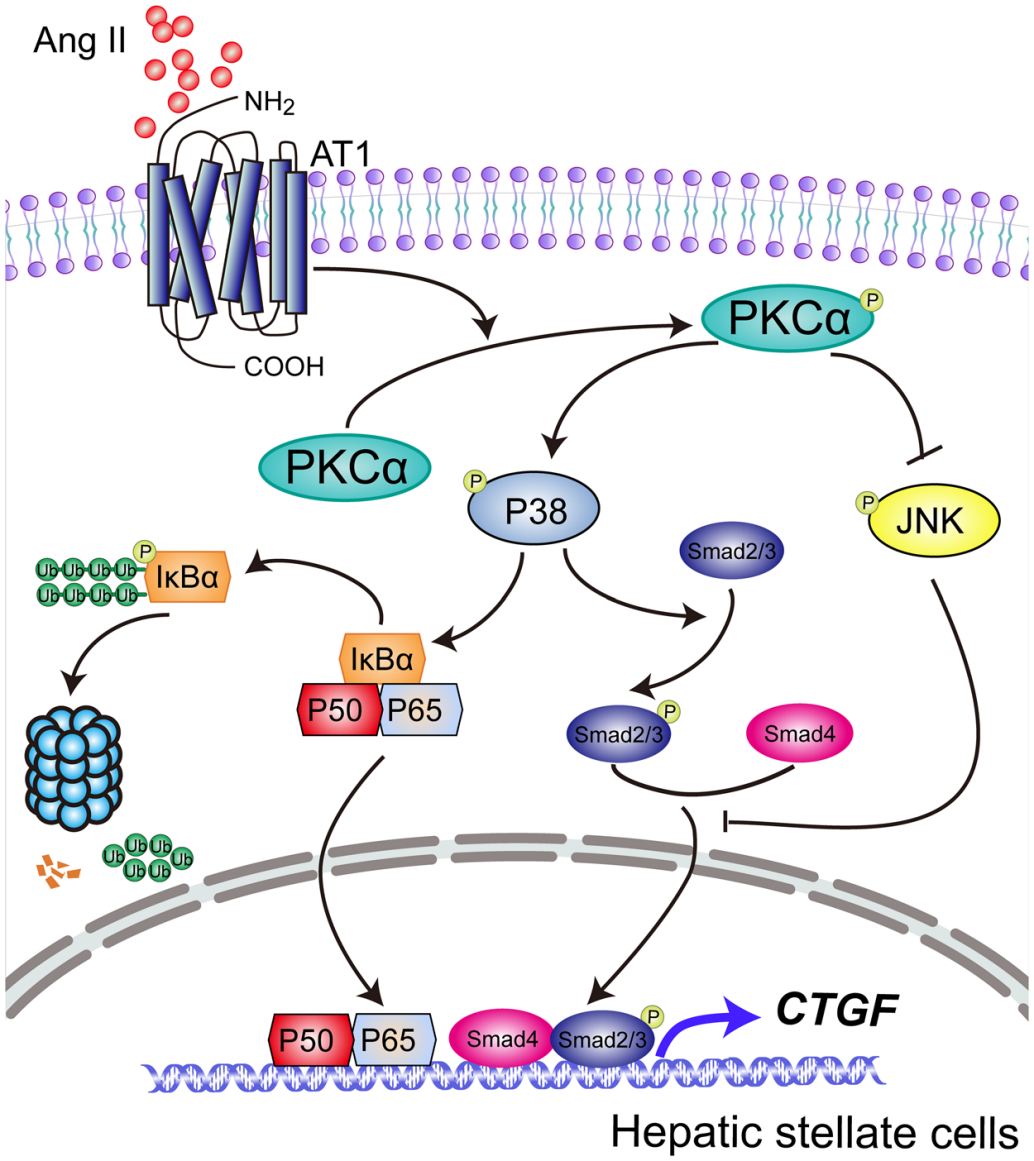
Schematic diagram of possible molecular mechanisms underlying the transcriptional regulation of CTGF expression in Ang II-stimulated HSCs.

In summary, the present studies demonstrate that Ang II induces CTGF expression and ECM synthesis in human HSCs through a unique mode of interaction between the NF-*κ*B and Smad2/3 signals activated by AT_1_/PKC*α*/p38 MAPK pathway (Fig. 10). In addition, Ang II inhibits JNK activity, which also facilitates the activation of Smad2/3 signaling. Our findings suggest that CTGF could participate in Ang II-mediated profibrotic effects in chronic liver diseases and might open up a new strategy to prevent pathological CTGF production in hepatic fibrosis.

## Materials and Methods

### Materials

Human angiotenin II (Ang II; MW: 1046.18), losartan (an AT_1_ receptor antagonist), chelerythrine chloride (CHE; a PKC inhibitor), SIS3 (a Smad3 inhibitor) and PI were purchased from Sigma (St. Louis, MO, USA). Antibodies against the following proteins were used: phospho-ERK1/2(Thr202/Tyr204), phospho-JNK(Thr183/Tyr185), phospho-p38 MAPK(Thr180/Tyr182), phospho- Smad3(Ser423/Ser425), phospho-Smad2(Ser465/Ser467)/Smad3(Ser423/Ser425), ERK1/2, SAPK/JNK, p38 MAPK, Smad2/3, and NF-*κ*B p65 (Cell Signaling Technology Inc., Beverly, MA, USA); TGF-*β*1 and phospho-I*κ*B*α*(Ser32/Ser36) (Signalway Antibody, Pearland, TX, USA); AT_1_, AT_2_, PKC-*α*, I*κ*B*α*, Smad4, lamin B1, MAPK phosphatase 7 (MKP7) and *β*-actin (Santa Cruz Biotechnology Inc., Santa Cruz, CA, USA); phospho-PKC-*α* (Thr497), PKC-*δ*, *ε*, and *ζ* (Abcam, Cambridge, MA, USA); CTGF (GeneTex Inc., Irvine, CA, USA); fibronectin (Bioworld Technology, Minneapolis, MN, USA); collagen type I (Boster biological technology, Wuhan, China). Fluorescein isothiocyanate (FITC)-conjugated goat anti-rabbit IgG, horseradish peroxidase (HRP)-conjugated goat anti-mouse and anti-rabbit IgG were obtained from Beijing Zhongshan Golden Bridge Biotechnology Co. Ltd (Beijing, China). Phorbol-12-myristate-13-acetate (PMA; a strong activator of PKC), SB203580 (a p38 MAPK inhibitor), and SP600125 (a JNK inhibitor) were purchased from Beyotime Institute of Biotechnology (Haimen, Jiangsu, China). U0126 (an inhibitor of MEK1/2) was provided by Cell Signaling Technology. SB-431542 (an inhibitor of TGF-*β* type I receptor) was obtained from Cayman Chemicals (Ann Arbor, MI, USA). BAY11-7082 (a NF-*κ*B inhibitor) and PD123319 (an AT_2_ receptor antagonist) were provided from Selleck Chemicals (Houston, TX, USA). All the other chemicals were purchased from Sigma unless otherwise indicated.

### Cell culture

LX-2, an immortalized human hepatic stellate cell line, was purchased from China Center for Type Culture Collection (CCTCC, Wuhan, China) and routinely cultured in Dulbecco’s modified Eagle’s medium (DMEM; Gibco, Carlsbad, CA, USA) supplemented with 10% fetal bovine serum (FBS; Gibco, Grand Island, NY, USA), 100 units/mL penicillin G, and 100 μg/mL streptomycin. The cell line was cultured at 37 °C in a CO_2_ incubator with a 95% air/5% CO_2_ humidified atmosphere. The culture medium was changed every other day.

### Quantitative reverse-transcription polymerase chain reaction (qRT-PCR)

Total RNA was isolated from unstimulated control or Ang II-stimulated cell pellets using a RNApure high-purity total RNA rapid extraction kit (spin-column, BioTeke, Beijing, China) according to the manufacturer’s protocols. Equal amounts of total RNA (2 μg) were reverse-transcribed using a BioTeke Super RT kit in 20 μl reaction volume. Quantitative real-time PCR was carried out on a CFX-96 thermocycler (Bio-Rad) using SYBR FAST qPCR Master Mix (Kapa Biosystems, Woburn, MA, USA) under the following conditions: 95 °C for 3 min, 40 cycles at 95 °C for 3 sec, 60 °C for 40 sec. The primers of CTGF: 5′-TCACTGACCTGCCTGTAG (forward), 5′-GCTGAGTCTGCTGTTCTG (reverse); TGF-*β*1: 5′-CCTCCTCCTGCCTGTCTG (forward), 5′-GTGTTGCTATGGTGACTGAATG (reverse); *β*-actin: 5′-GTCCACCGCAAATGCTTCTA (forward), 5′-TGCTGTCACCTTCACCGTTC (reverse) were synthesized by Invitrogen Life Technologies (Shanghai, China). Fluorescence signal was acquired at the end of the elongation step (60 °C for 40 sec) of every PCR cycle in order to track the increase in amount of amplified DNA. Melt curve was obtained by heating the amplicon from 60 to 95 °C to ensure purity of PCR products. For each assay, a standard curve from consecutive 5-fold dilutions of a cDNA pool representative of all samples was constructed to verify qPCR efficiency (90–110%). The threshold cycle (Ct) is defined as the fractional PCR cycle number at which the fluorescence passes the fixed threshold. The Ct is inversely proportional to the logarithm of the initial amount of template in the PCR. Fold changes in mRNA expression were calculated by the comparative Ct method (2^−ΔΔCt^)^44^, using *β*-actin as the housekeeping gene.

### Assay of PKC activity

LX-2 cells were seeded into 100-mm dishes at a density of 5 × 10^5^ cells/dish. When cells were grown to 70% confluence, they were synchronized by serum starvation for 18 h, then the cells were incubated with Ang II (10^−7^ M; dissolved in DMEM culture medium without FBS) for various periods of time. To determine PKC cell membrane translocation activity, cytosolic and membrane fractions were prepared using a Mem-PER^TM^ Plus Membrane Protein Extraction Kit (Thermo Fisher Scientific Inc., Rockford, IL, USA) according to the manufacturer’s instructions. The PKC kinase activity in crude cytosolic and membrane preparations was measured in quadruplicate on microtiter plates by a commercially available kit (Enzo Life Sciences Inc., Farmingdale, NY, USA), which is based on a solid-phase enzyme-linked immunosorbent assay (ELISA) that uses a specific peptide substrate for PKC and a specific antibody that reacts with the phosphorylated substrate. Protein concentration in the cytoplasmic and membrane fractions was measured with a bicinchoninic acid (BCA) protein assay kit (Beyotime). The PKC activity was finally corrected for protein content.

### Immunofluorescence staining

LX-2 cells were seeded at a density of 1 × 10^4^ cells/mL on coverslips in 24-well plates and then treated under the indicated conditions. After three washes with phosphate-buffered saline (PBS), cells were fixed in 4% paraformaldehyde for 0.5 h, permeabilized using 0.3% (v/v) Triton X-100 for 0.5 h, washed 3 times with PBS, and blocked with 10% normal sheep serum in PBS for 1 h at room temperature; then incubated with primary antibodies against PKC-*α* (1:100 dilution), phospho-Smad2/3 (1:200), and NF-*κ*B p65 (1:100) at 4 °C overnight. Cells were then incubated with FITC-labeled secondary antibodies (1:50 dilution) for 2 h at room temperature. To visualize cell nuclei, cells were counterstained with propidium iodide (PI) for 2 min at room temperature. Finally, stained cells were scanned and photographed with a Leica TCS-SP2 confocal laser scanning microscope (Leica Microsystems, Wetzlar, Germany).

### EMSA

Nuclear lysates of LX-2 cells were prepared using a NE-PER^TM^ Nuclear and Cytoplasmic Extraction Kit (Thermo). The DNA binding activities of NF-*κ*B and phospho-Smad3 in nuclear extracts were assessed by a Chemiluminescent EMSA Kit (Beyotime) according to the manufacturer’s instructions. Briefly, Nuclear extracts (5 μg) from LX-2 cells were preincubated in 5× binding buffer for 10 min at room temperature, and then incubated for an additional 20 min at room temperature with 2 pmol of biotin-labeled double-stranded oligonucleotide probes containing NF-*κ*B binding sites (5′-AGTTGAGGGGACTTTCCCAGGC-3′, consensus sequences underlined, Beyotime) and Smad3/4 binding sites (5′-CGAGCTTTTTCAGACGGAGGAATGCTGAGTGTCA-3′, consensus sequences underlined; SBS Genetech Co., Ltd., Beijing, China) in 20 μL of reaction mixture. To examine the specificity of binding of transcription factor to DNA, nuclear extracts were preincubated with either 50-fold molar excess of wild-type or mutated unlabeled consensus oligonucleotide competitor at room temperature for 0.5 h. The following mutated unlabeled sequences were used: NF-*κ*B (5′-AGTTGAGGCGACTTTCCCAGG-3′), and Smad3/4 (5′-CGAGCTTTTTCAGATAGAGGAATGCTGAGTGTCA-3′). For supershift analysis, nuclear extracts were incubated with a rabbit monoclonal antibody (2 μg) against either NF-*κ*B p65 or phosphorylated Smad3 in binding buffer for 1 h at room temperature before addition of the labeled oligonucleotide probes. The DNA-protein complexes were separated electrophoretically on a 6% nondenaturing polyacrylamide gel in 0.5× Tris-borate-EDTA buffer for 1 h, and then transferred to a positively charged nylon membrane. The membranes were UV cross-linked for 5 min. Finally, the biotin-labeled DNA bands were visualized using an enhanced chemiluminescence (ECL) assay kit (Millipore, Bedford, MA, USA).

### Transient cell transfections and luciferase reporter assays

LX-2 cells were grown in 24-well plates for 24 h and then transiently transfected with a 200 ng of luciferase reporter plasmid containing either multiple NF-*κ*B response elements (pNF-KB-Luc; Beyotime) or four tandem Smad-binding elements (pSBE4-Luc; Addgene, Cambridge, MA, USA). In each well, cells were also co-transfected with a 5 ng of pRL-TK control plasmid (Promega, Madison, WI, USA), encoding Renilla luciferase gene for normalizing transfection efficiency. An empty pGL6-Luc or pBV-Luc plasmid was transfected into cells to provide a control for basal expression of the luciferase reporter gene. Lipofectamine 2000™ reagent (Life Technologies Inc., Carlsbad, CA, USA) was used for tranfection. After 40 h of incubation, cells were subjected to Ang II (10^−7^ M) stimulation for a further 0.5 h. Subsequently, cell lysates were prepared, and luciferase activities were measured fluorimetrically using Dual-Luciferase Reporter Assay kits (Promega) in a Thermo Varioskan Flash Luminometer (Thermo).

### Determination of CTGF level by ELISA

LX-2 cells (5 × 10^5^ cells/well) were cultured in 6-well plates overnight and synchronized using serum starvation for 18 h. After starvation, cells were pretreated with different inhibitors for 4 h, and stimulated with Ang II (10^−7^ M) for another 4 h. CTGF concentrations in culture supernatants were assayed using a human CTGF ELISA Kit (Peprotech, Rocky Hill, NJ, USA) according to the manufacturer’s recommendations. The final CTGF concentration was calculated by a standard optical density concentration curve within the range of 63–4000 pg/mL. Data are expressed as fold induction relative to the vehicle-treated cells.

### RNA interference

For inhibition of TGF-*β*1, MKP7 or CTGF expression, LX-2 cells in 6-well plates (5 × 10^4^ cells/well) were transfected with TGF-*β*1 siRNA (Santa Cruz) or Trilencer-27 siRNA duplexes against MKP7 or CTGF (Origene Technologies Inc., Rockville, MD, USA) using Lipofectamine RNAiMAX Reagent (Life Technologies Inc.) according to the instructions of the manufacturer. Transfection using universal scrambled negative control siRNA duplex (Origene) was used as a negative control. After transfection for 48 h, LX-2 cells were exposed to Ang II (10^−7^ M) for another 0.5, 1, 4, or 24 h, respectively. Transient siRNA-mediated attenuation of TGF-*β*1, MKP7 or CTGF expression was determined by immunoblotting analysis with the respective antibodies.

### Immunoblotting analysis

Subcellular fractionation was carried out as we reported previously^44^. After determining protein concentration using the BCA method (Beyotime), equal amounts of protein lysates (40 μg) were separated by 8–12% sodium dodecyl sulfate-polyacrylamide gel electrophoresis (SDS-PAGE). Resolved proteins were transferred to polyvinylidene difluoride (PVDF) membranes (Immobilon-P, Millipore) in a transfer buffer containing 25 mM Tris-HCl (pH 8.3), 192 mM glycine and 20% (v/v) methanol. After nonspecific binding sites were blocked with 5% nonfat dried milk in Tris-buffered saline (20 mM Tris, pH 7.6, 150 mmol/L NaCl) containing 0.05% (v/v) Tween 20 (TBS-T) for 1 h at room temperature, membranes was incubated overnight at 4 °C with the indicated primary antibodies, followed by incubation for 1 h at room temperature with a 1:3000 dilution of a secondary horseradish peroxidase-conjugated anti-rabbit or anti-mouse IgG. Immune-reaction bands were visualized by the ECL reagent (Millipore) and detected by Gel Doc/Chemi Doc Imaging System (Bio-Rad). Equal loading of the gel was confirmed by probing for *β*-actin with similar results. The signal intensities of bands, expressed as arbitrary densitometric units, were quantified by Quantity One software (Bio-Rad). Results are presented as a ratio of phosphorylated/activated protein to total/housekeeping protein and normalized to the same ratio for the corresponding control sample, which is assigned a value of 1.0.

### Statistical analysis

Data from 3 biological replicates were averaged for the analysis. All data are expressed as means ± standard deviation (SD). Inter-group differences were compared by one-way analysis of variance (ANOVA); when differences were significant by this test (*P* < 0.05), multiple comparisons were assessed by Tukey post hoc test. All statistical analyses were carried out with SPSS version 19.0 Statistical Software (IBM, Chicago, IL, USA). In all cases, a *P* < 0.05 was considered to be statistically significant.

## Electronic supplementary material

Supplemental Figures
