# Supplementary material for: Angiotensin II induces connective tissue growth factor expression in human hepatic stellate cells by a transforming growth factor β-independent mechanism
Source: Sci Rep. 2017 Aug 10;7:7841. doi: 10.1038/s41598-017-08334-x (PMC5552744; doi:10.1038/s41598-017-08334-x)
Supplement: Supplementary file 1 — Supplemental Figures [file 41598_2017_8334_MOESM1_ESM.pdf]

Angiotensin II induces connective tissue growth factor expression in human hepatic stellate cells by a transforming growth factor *β*-independent mechanism

Ao Li[1,2,*], Jingyao Zhang[1,*], Xiaoxun Zhang[1,*], Jun Wang[1], Songsong Wang[2], Xiao Xiao[1], Rui Wang[1], Peng Li[2], Yitao Wang[2]

[1]College of Pharmacy and Bioengineering, Chongqing University of Technology, Chongqing 400054, China.

[2]State Key Laboratory of Quality Research in Chinese Medicine, Institute of Chinese Medical Sciences, University of Macau, Macau 999078, China

[*]These authors contributed equally to this work.

Correspondence and requests for materials should be addressed to R. W. (email: wangrx1022@163.com) or P. L. (email: pli1978@hotmail.com).

Supplemental Figure S1

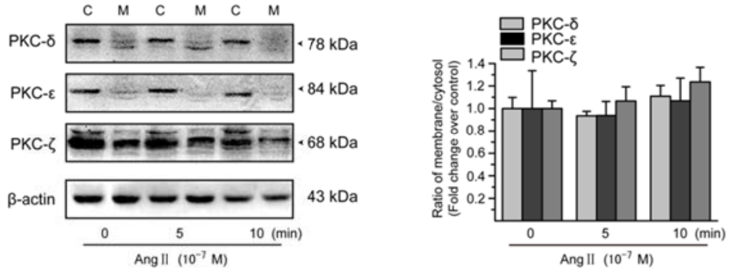

Figure S1. Effects of Ang II on PKCδ, ε, and ζ translocation in LX-2 cells. Serum-starved LX-2 cells were treated with Ang II (10-7 M) for the indicated time points (0, 5, and 10 min). The cytosolic (C) and membrane (M) fractions were prepared and subjected by immunoblotting with the indicated antibodies. β-Actin served as an internal control. Similar results were observed in 3 independent experiments, and representative immunoblots for each protein are shown. The membrane-to-cytosol ratio was used to calculate fold translocation (or activation) over that of unstimulated cells (defined as 1-fold). Each bar represents mean ± SD of 3 independent experiments.

Supplemental Figure S2

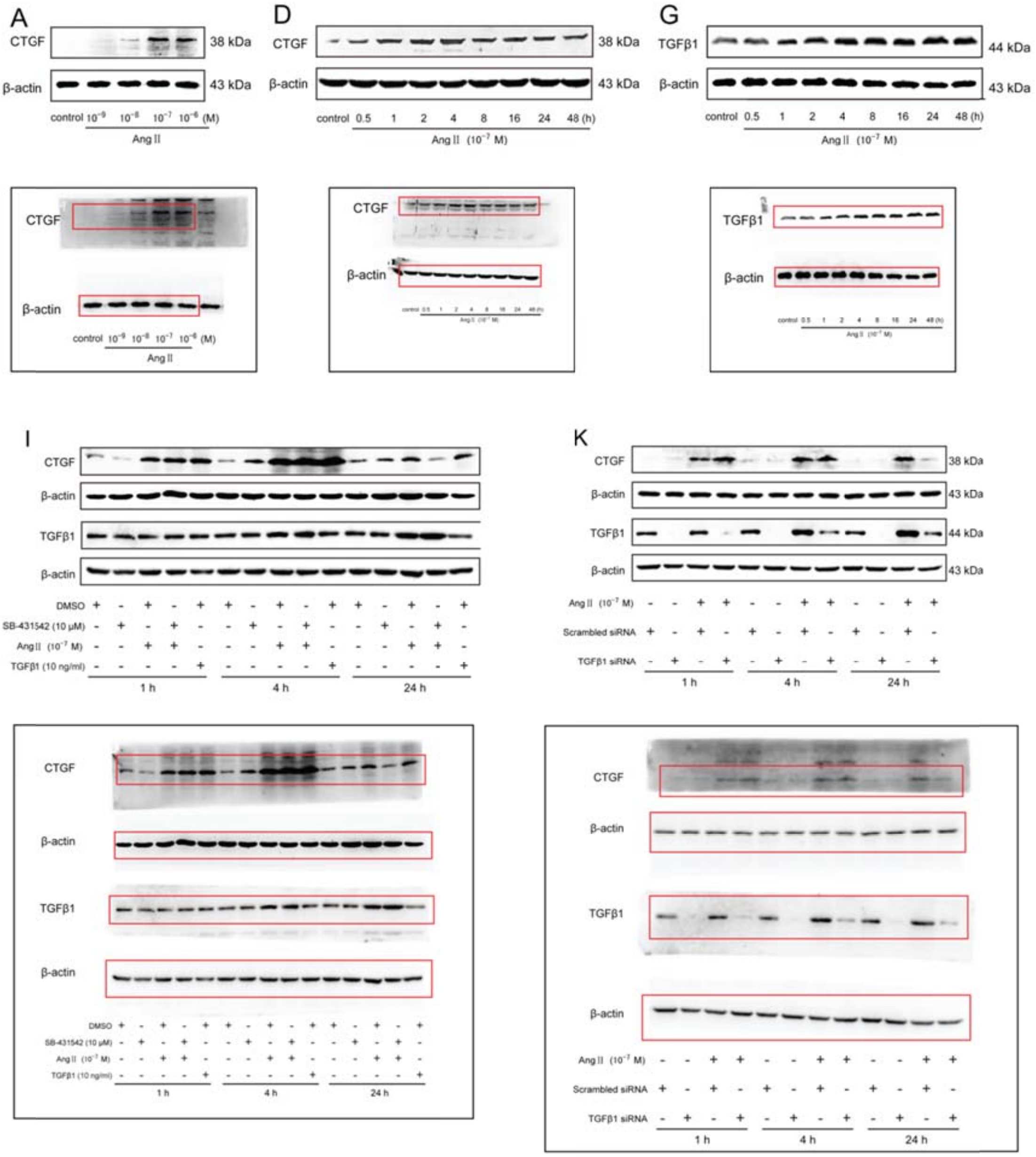

Figure S2. Full length blots of Figure 1. Red lines show the cropping locations.

Supplemental Figure S3

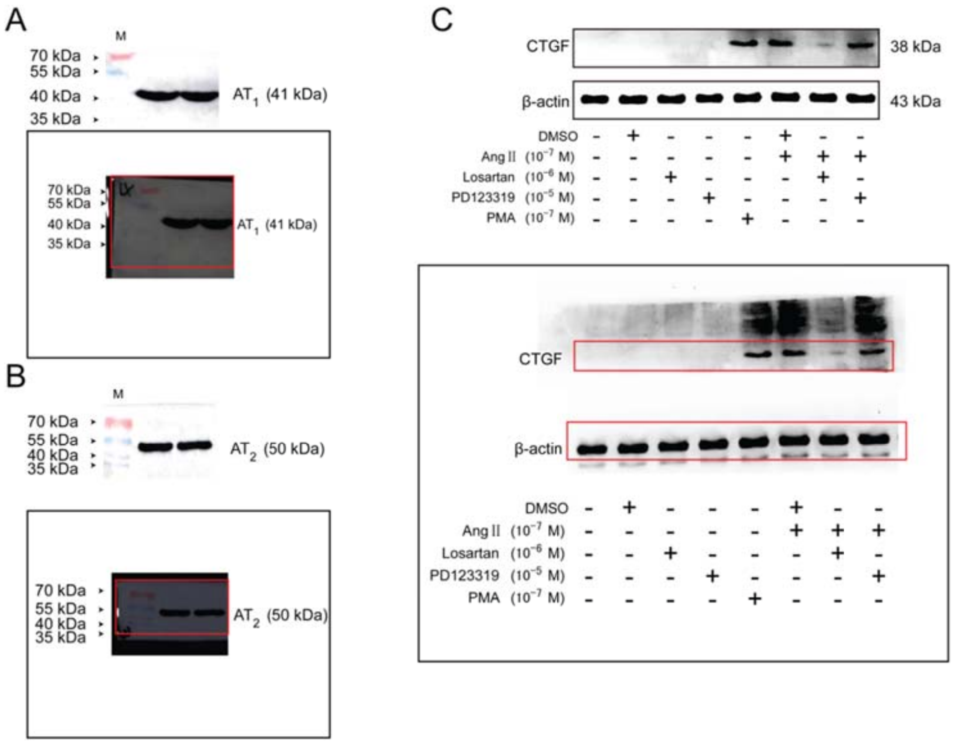

Figure S3. Full length blots of Figure 2. Red lines show the cropping locations.

Supplemental Figure S4

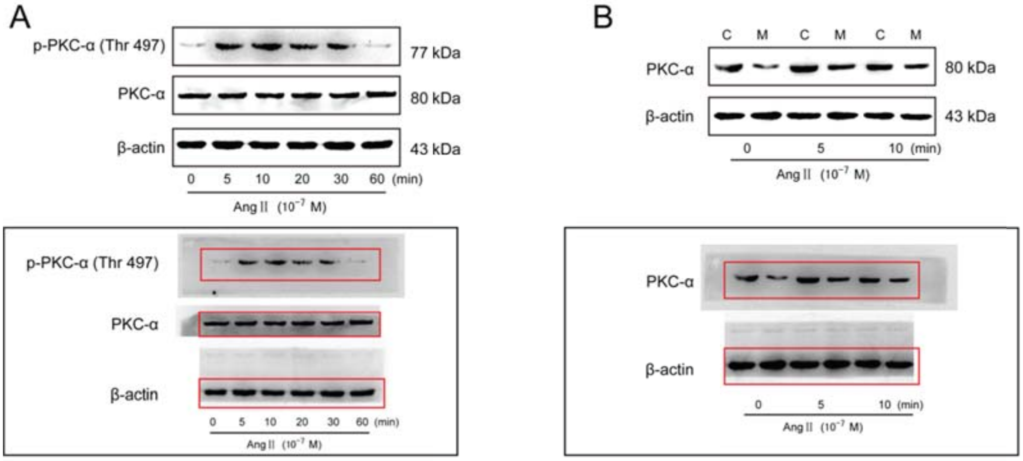

Figure S4. Full length blots of Figure 3. Red lines show the cropping locations.

Supplemental Figure S5

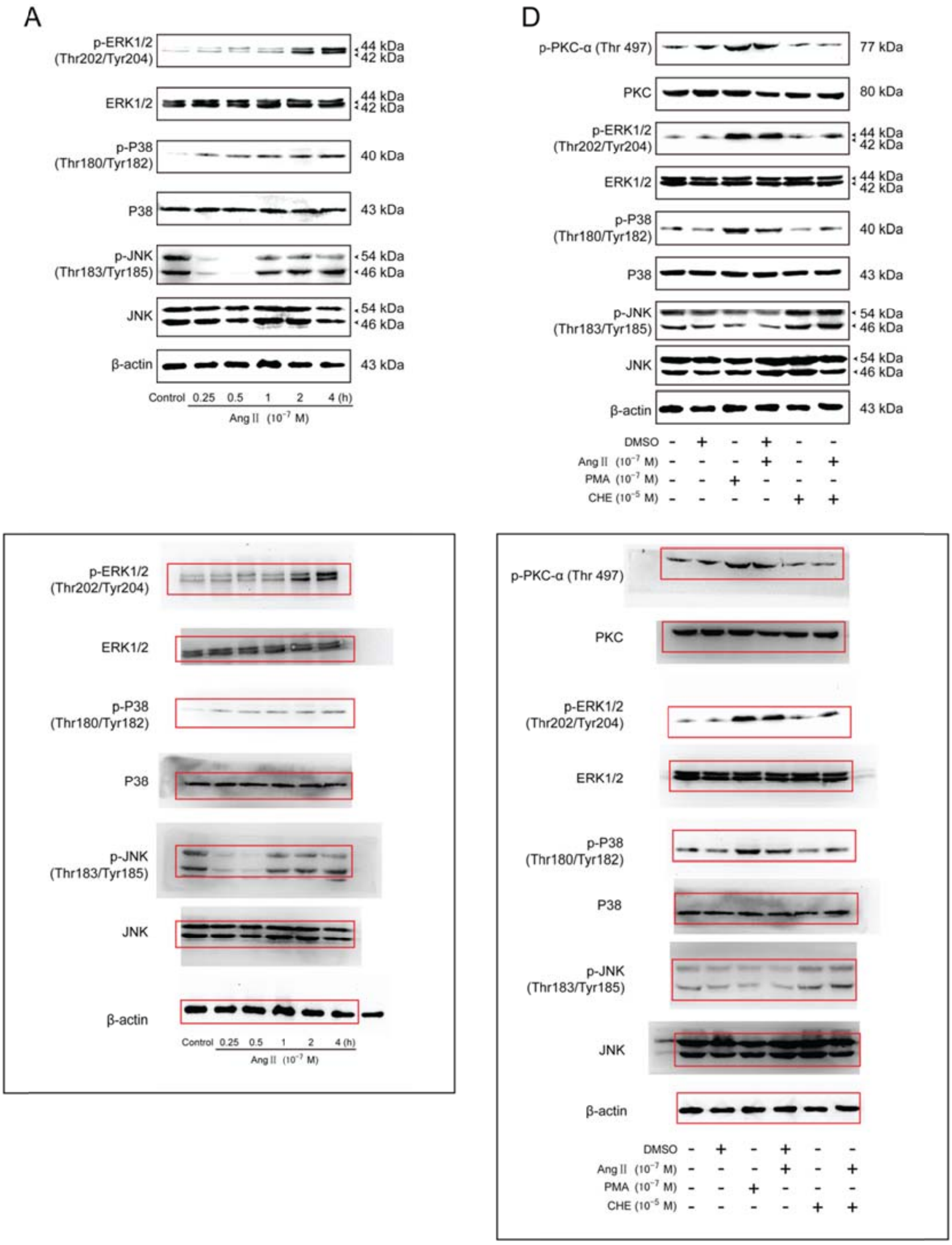

Figure S5. Full length blots of Figure 4. Red lines show the cropping locations.

Supplemental Figure S6

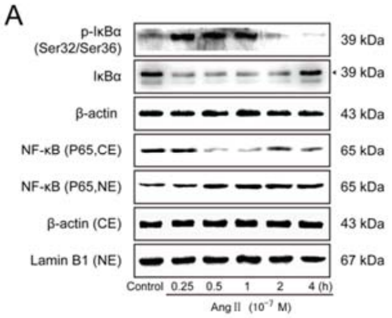

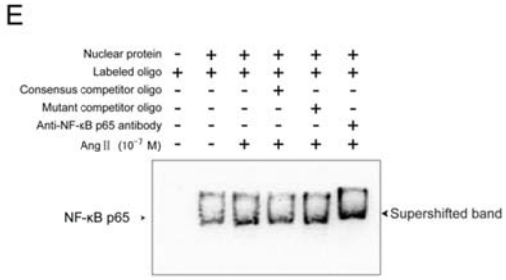

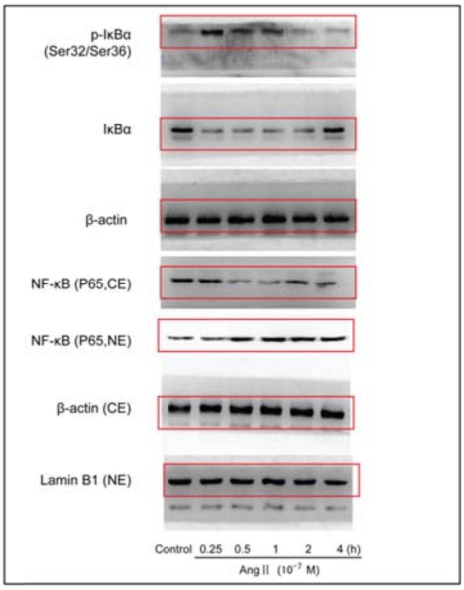

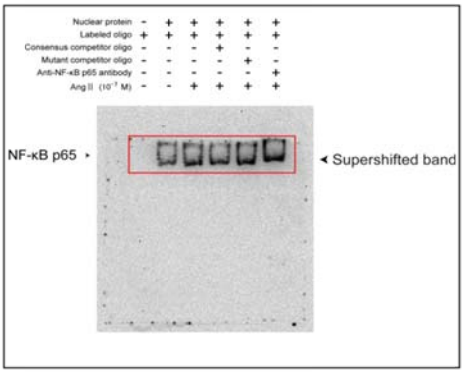

Figure S6. Full length blots of Figure 5. Red lines show the cropping locations.

Supplemental Figure S7

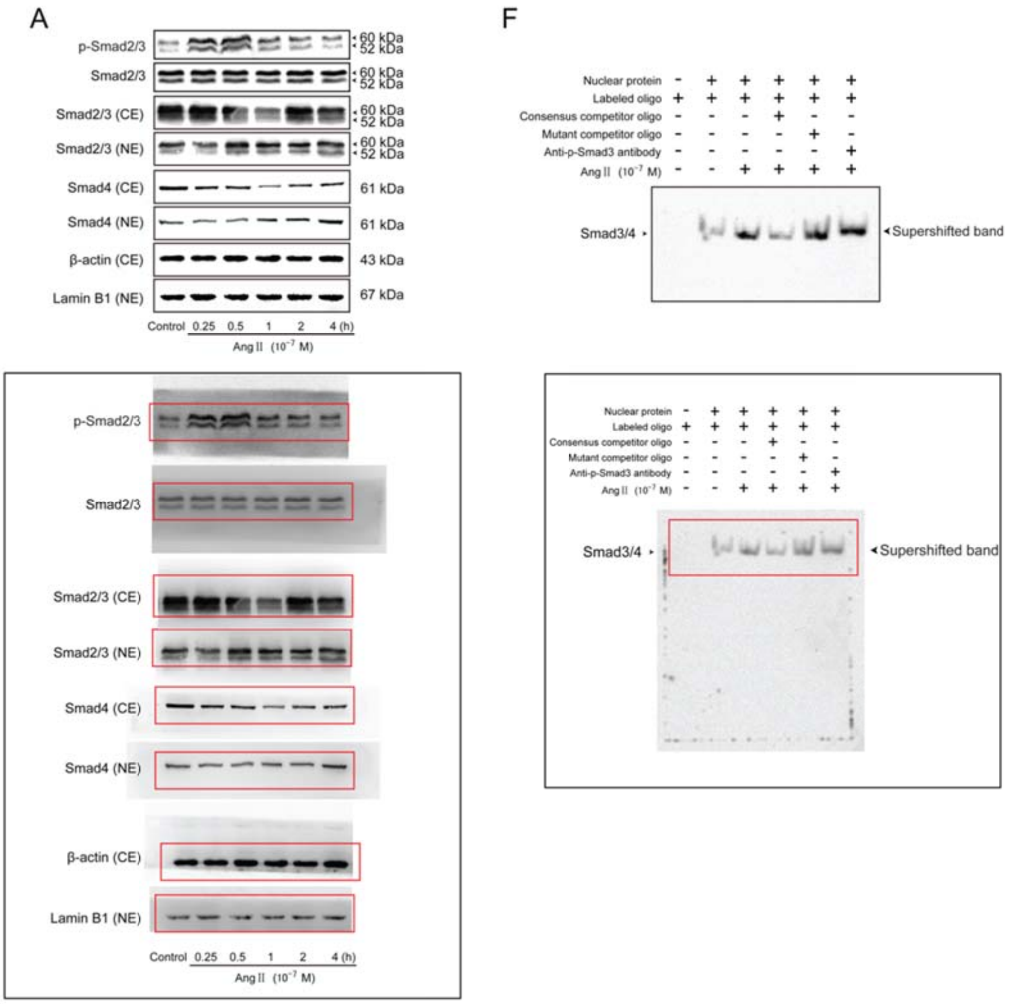

Figure S7. Full length blots of Figure 6. Red lines show the cropping locations.

Supplemental Figure S8

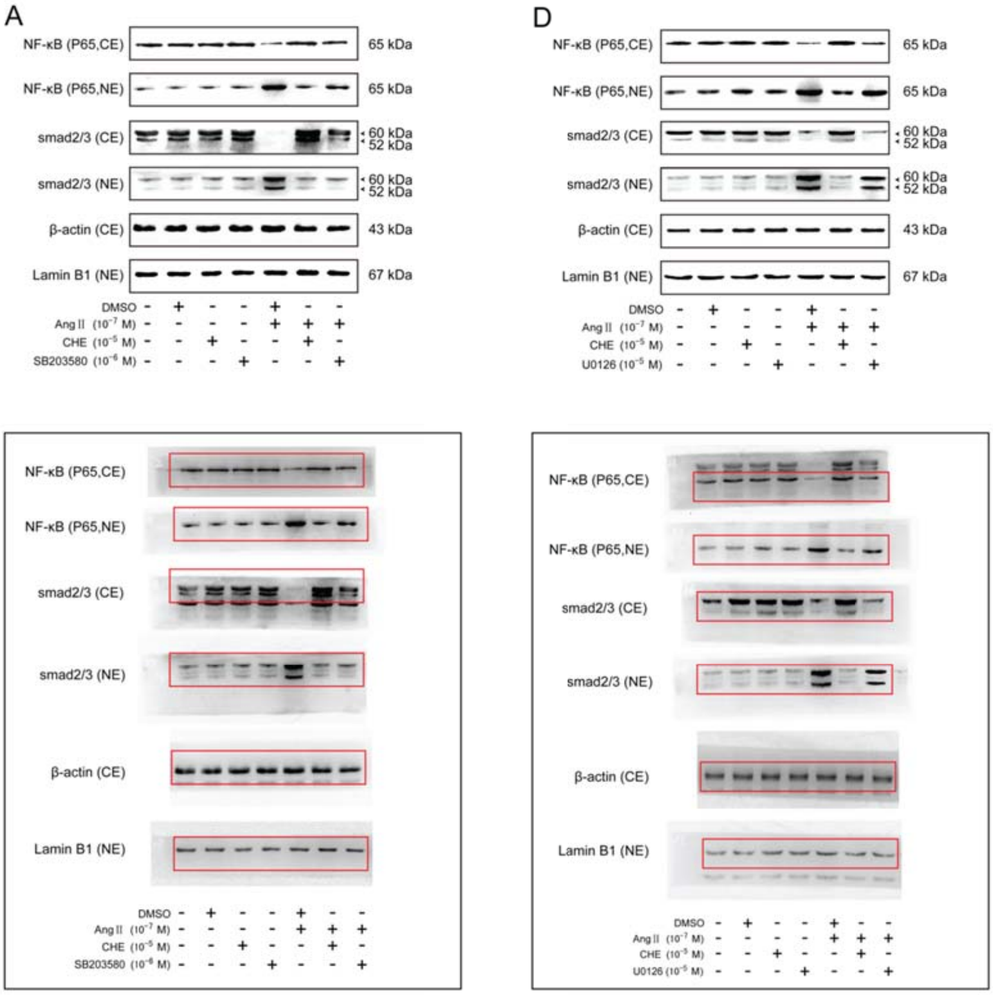

Figure S8. Full length blots of Figure 7. Red lines show the cropping locations.

Supplemental Figure S9

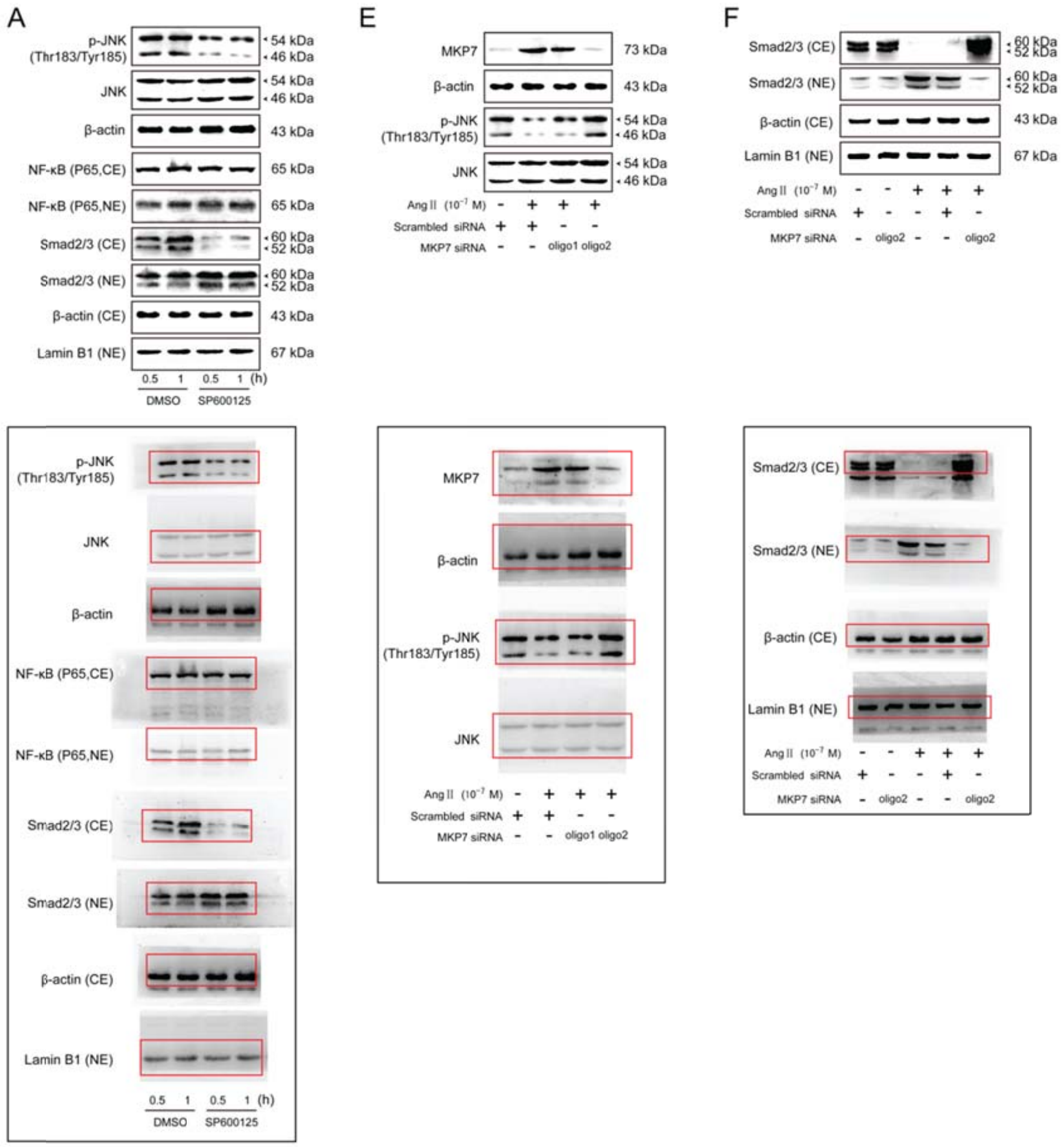

Figure S9. Full length blots of Figure 8. Red lines show the cropping locations.

Supplemental Figure S10

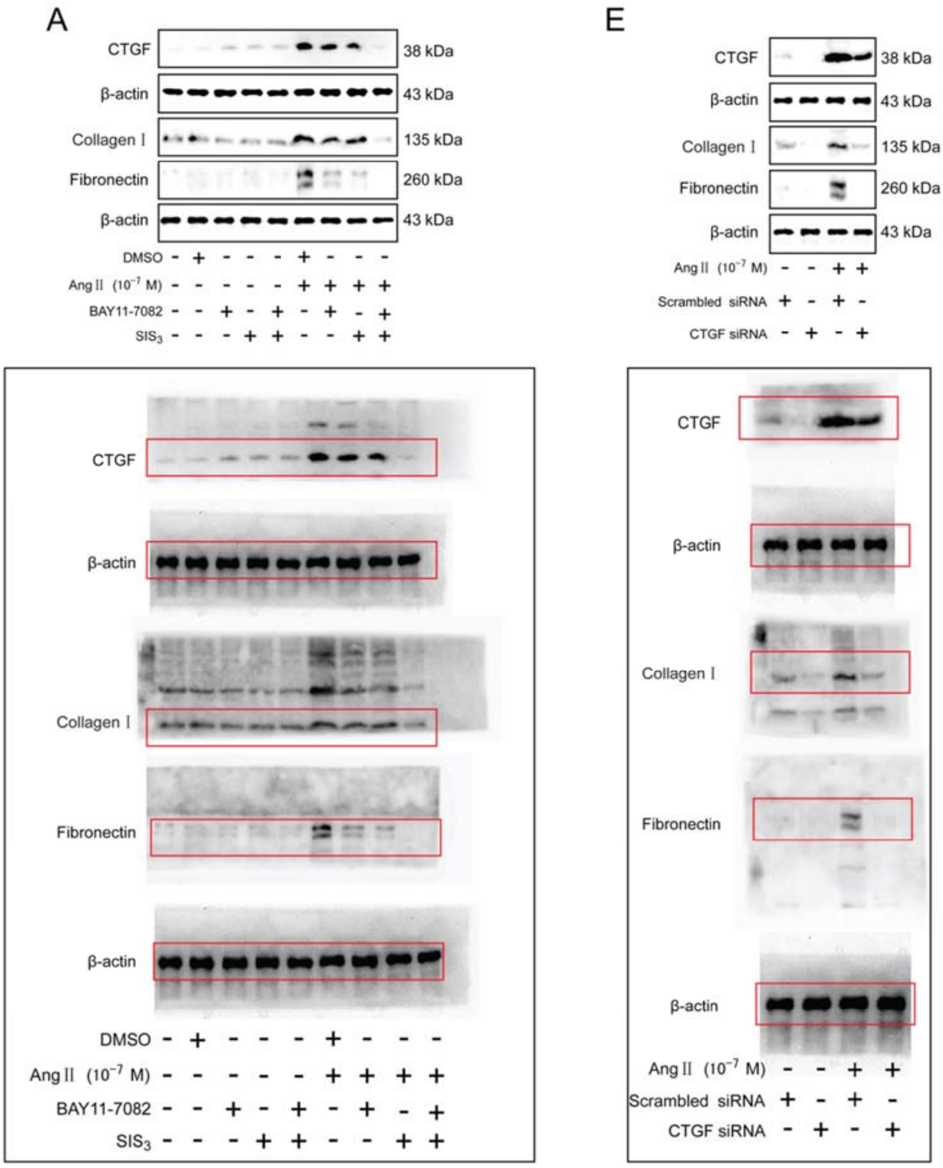

Figure S10. Full length blots of Figure 9. Red lines show the cropping locations.

Supplemental Figure S11

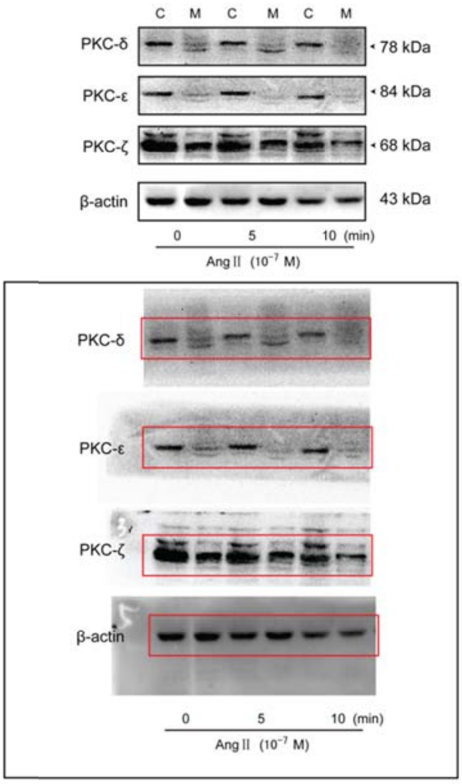

Figure S11. Full length blots of Figure S1. Red lines show the cropping locations.
